# The value of health—Empirical issues when estimating the monetary value of a quality‐adjusted life year based on well‐being data

**DOI:** 10.1002/hec.4279

**Published:** 2021-05-05

**Authors:** Sebastian Himmler, Jannis Stöckel, Job van Exel, Werner B. F. Brouwer

**Affiliations:** ^1^ Erasmus School of Health Policy & Management Erasmus University Rotterdam Netherlands; ^2^ Erasmus School of Economics Erasmus University Rotterdam Netherlands

**Keywords:** health valuation, instrumental variable regression, panel data, piecewise regression, quality‐adjusted life years, well‐being valuation

## Abstract

Decisions on interventions or policy alternatives affecting health can be informed by economic evaluations, like cost‐benefit or cost‐utility analyses. In this context, there is a need for valid estimates of the monetary equivalent value of health (gains), which are often expressed in € per quality‐adjusted life years (QALYs). Obtaining such estimates remains methodologically challenging, with a recent addition to the health economists' toolbox, which is based on well‐being data: The well‐being valuation approach. Using general population panel data from Germany, we put this approach to the test by investigating several empirical and conceptual challenges, such as the appropriate functional specification of income utility, the choice of health utility tariffs, or the health state dependence of consumption utility. Depending on specification, the bulk of estimated € per QALY values ranged from €20,000–60,000, with certain specifications leading to more considerable deviations, underlining persistent practical challenges when applying the well‐being valuation methodology to health and QALYs. Based on our findings, we formulate recommendations for future research and applications.

## 1 INTRODUCTION

During the ongoing COVID‐19 pandemic, many citizens for the first time directly observe scarcity of goods in the health care sector in terms of testing, ventilation, vaccination capacity, and the prioritisation of services under binding capacity constraints. This scarcity and the broader societal consequences of the pandemic has revealed many difficult trade‐offs between health and the economy, and between the needs of different patient groups within the health care sector. While the current attention to such matters is unprecedented, policy makers are confronted with many of these trade‐offs also in non‐pandemic times. To make informed decisions on policy options, however, requires decision makers to weigh up health and economic consequences, aiming to ensure maximum benefit or minimal harm. Welfare economic tools like cost‐benefit‐analysis can aid decision makers in this process by providing relevant and clear information to openly address the nature of the trade‐offs being made (Chilton et al., 2020; Donaldson & Mitton, 2020; Hendren & Sprung‐Keyser, 2020).

Cost‐benefit analyses entail measuring and valuing gains and losses (benefits and costs) in monetary units, thereby allowing a holistic perspective on societal trade‐offs and identifying which policy option is socially most preferred. In the context of interventions and policies affecting population health (though not necessarily aimed primarily at health), cost‐benefit analysis therefore requires to obtain estimates on the monetary equivalent value of health, from here onwards denoted as *v*_*Q*_ (McIntosh, 2010).

In the narrow health care context, *v*_*Q*_, depending on the jurisdiction (Rowen et al., 2017), constitutes an important parameter in health technology assessment. There, value for money considerations are often operationalized using cost‐utility analysis, where a new technology's costs are compared to its expected health gain, measured using Quality Adjusted Life Years (QALYs) (Neumann et al., 2016). Equation (1) formulates a generalisation of the corresponding decision rule, with Δ*Q* denoting the health gain (in QALYs) and Δ*c*_*t*_ the total costs compared to the alternative treatment:

(1) $$\frac{\Delta c_{t}}{\Delta Q}<v_{Q}$$

This cost‐effectiveness ratio (ICER) is acceptable, if it lies below *v*_*Q*_ corresponding to one QALY (Brouwer et al., 2019).^1^ While the use and empirical foundation of such threshold values within health care vary across jurisdictions (Cameron et al., 2018; Cleemput et al., 2011), estimating the level of *v*_*Q*_ corresponding to one QALY, also for the purpose of cost‐benefit analysis, is challenging and has been attempted using various methods (see background section). In this endeavour, Huang et al. (2018) were the first to conceptualize and apply the well‐being valuation approach for estimating a QALY equivalent *v*_*Q*_, providing estimates of A$42,000 (€28,000) to A$67,000 (€45,000). This method is based on the marginal rate of substitution between income and health. Further exploration of the approach is needed to be able to judge whether the corresponding estimates are indeed helpful for informing *v*_*Q*_. This paper aims to make the following contributions: Firstly, by applying a similar approach as Huang et al. (2018) and using data from a different context, we generate further insights regarding the validity and reliability of the well‐being valuation method for determining *v*_*Q*_. Secondly, we aim to address some empirical and methodological challenges associated with applying the well‐being valuation method in general and for valuing QALYs in particular, which were not fully addressed in previous studies. By using German data, an additional contribution lies in providing information on *v*_*Q*_ for a context in which such estimates are scarce, a result of German health authorities not (explicitly) basing their reimbursement decisions on the framework outlined in Equation (1).^2^

We used data from the Socio‐Economic Panel (2019), or SOEP, from 2002 to 2018. Fixed‐effects and instrumental variable regressions were used to address endogeneity concerns regarding the impact of income on life satisfaction. Our baseline estimates indicate population average monetary valuations of a QALY of €22,717 and €58,533, with and without instrumenting for income. However, alternative specifications and robustness checks lead to varying estimates, highlighting the empirical challenges and the consequences of methodological choices on the obtained monetary values, and areas for future research.

## 2 THE SEARCH FOR *v*_*Q*_ AND THE WELL‐BEING VALUATION METHOD

Various methods have been used in the ongoing endeavour of obtaining estimates of *v*_*Q*_, producing a range of conceptually different values. One approach, employed by Mason et al. (2009), bases *v*_*Q*_ on estimates of the value of preventing a statistical fatality, a concept commonly used in public sector safety policies. Another approach calculating *v*_*Q*_ entails using relative risk aversion in relation to income (Phelps, 2019). However, *v*_*Q*_ estimates have predominantly been obtained based on stated preferences, by asking individuals directly about their willingness to pay (WTP) for specific health gains. Ryen and Svensson (2015) summarized the extensive literature that used WTP methods to identify *v*_*Q*_ and reported trimmed mean and median estimates of €74,159 and €24,226 (in 2010 price levels).

Huang et al. (2018) proposed an alternative method for estimating *v*_*Q*_, based on revealed, although subjective, information: the well‐being valuation approach. This method has been applied to obtain monetary valuations for various other non‐market goods, including specific health outcomes and diseases (Brown, 2015; Ferrer‐i‐Carbonell & van Praag, 2002; Howley, 2017; McNamee & Mendolia, 2018), informal care provision (Mcdonald & Powdthavee, 2018; van den Berg & Ferrer‐i‐Carbonell, 2007), air pollution and natural disasters (Luechinger, 2009; Luechinger & Raschky, 2009), national security (Frey et al., 2009) or the welfare effects of sports events (Dolan et al., 2019). In their study, Huang et al. (2018) used data from the HILDA panel survey from Australia and obtained *v*_*Q*_ estimates of A$42,000 (€28,000) to A$67,000 (€45,000), which were similar to threshold values applied for funding decisions in Australia. Recently, Himmler et al. (2020) applied the well‐being valuation approach in a cross‐sectional sample from the UK to estimate *v*_*Q*_, as well as an equivalent value for broader well‐being. They report a base case *v*_*Q*_ estimate of £30,786 (approximately €35,000).

Both stated preference WTP and well‐being valuation approaches have advantages and disadvantages and may answer different questions based on how *v*_*Q*_ is specified. The former allows researchers to tailor their experimental design to specific contexts and control for undesired influences. For instance, WTP can be expressed from an individual or societal perspective (Bobinac et al., 2013), capturing more than self‐interested motivations when establishing WTP‐based *v*_*Q*_ estimates. Similarly, equity concerns relating to specific health states or streams (Dolan & Olsen, 2001; Pinto‐Prades et al., 2014), but also socio‐economic health inequalities can be connected with the QALY framework (Wagstaff, 1991). Furthermore, one can also pose WTP questions from an *ex‐ante* or *ex‐post* perspective, with the former having the advantage of capturing options value (Gyrd‐Hansen, 2003; Philipson & Jena, 2006). However, the practice of asking individuals directly for the value of a prospect brings unique challenges; hypothetical response bias and insensitivity to scope or framing effects are only some of the practical concerns (see Kling et al. (2012)) that have been found to apply when obtaining WTP estimates for a QALY (Ahlert et al., 2016; Bobinac et al., 2012; Gyrd‐Hansen et al., 2014; Soeteman et al., 2017).

The well‐being valuation approach avoids these challenges by relying on (usually) large‐scale observational data, promising to provide a more inclusive picture of the range of preferences over health and wealth across diverse sub‐populations. However, the approach limits the scope to respondents' individual *ex‐post* valuations, while endogeneity concerns are a prevailing issue as it relies on the estimation of causal effects of health and income to calculate trade‐offs.

## 3 METHODS

### 3.1 Conceptual framework

We generally followed the framework proposed by Huang et al. (2018) for obtaining *v*_*Q*_. In a simplified model, the subjective well‐being (SWB) of individual *i* at time *t*, as a proxy for individual utility, is assumed to be described by:

(2) $$W_{it}=W\left(Y_{it},H_{it}\right)$$

where *W*_*it*_ is a vector of the individual's well‐being at all observed time points (*w*_*it*_), *Y*_*it*_ is the corresponding incomes (*y*_*it*_), and *H*_*it*_ a vector of health states (*h*_*it*_). The total well‐being experienced by individual *i* over a time interval of length *T* can then be described by a simple cumulative sum of individual well‐being states across time;

(3) $$W_{i}=\overset{T}{\underset{t=0}{\sum }}W\left(Y_{it},H_{it}\right)$$

Within this framework, consider an individual experiencing a change to their health vector Δ*H*_*i*_ within the time window *T*. For the individual to remain on the same level of SWB *W*_*i*_ requires an offsetting income change Δ*Y*_*i*_;

(4) $$W_{i}=W\left(Y_{i}+\Delta Y_{i},H_{i}+\Delta H_{i}\right)$$

The proposed approach estimates the population average Δ*Y* necessary to offset an imposed hypothetical health state change Δ*H* over *T* equivalent to one QALY. Therefore, Δ*Y* is the compensating income variation (CIV) for one QALY, or short *CIV*_*QALY*_.

### 3.2 Baseline specification

Following Huang et al. (2018), an ordinary least squares (OLS) fixed‐effects regression was estimated to calculate the impact of health and income on SWB within a time window *T* of 2 years (*t*_0_ and *t*_−1_). Modelling SWB as linear despite the cardinal nature of life satisfaction is a widely used approach, see for example, Ferrer‐i‐Carbonell and van Praag (2002). The underlying empirical model takes the following form;

(5) $$W_{irt}=\alpha +\beta_{0}H_{irt}+\beta_{1}H_{irt-1}+\delta_{0}Y_{irt}+\delta_{1}Y_{irt-1}+\tau X_{irt}+\lambda_{i}+\mu_{r}+\epsilon_{t}+u_{irt}$$

where *W*_*irt*_ refers to the SWB of individual *i* living in region *r* at time *t*, measured using life satisfaction data. The individual's health status *H*_*irt*_ is captured by health utility values based on the short form six dimensions (SF‐6D) instrument and its UK utility tariff (Brazier & Roberts, 2004). Household income is denoted by *Y*_*irt*_. Lagged variables of health and income were included to not be limited to short‐term one‐year changes and to partly account for reverse causality. We control for a vector *X*_*irt*_ of other potential time‐varying confounders. To account for time‐invariant unobservables, we incorporated individual (λ_*i*_), state (μ_*r*_), and time (ϵ_*t*_) fixed‐effects. *u*_*irt*_ denotes the error term. Heteroscedasticity‐robust standard errors were used in all estimations.

In a second step, we obtained *CIV*_*QALY*_ values by dividing the health status coefficients (β_0_ and β_1_) by the income coefficients (δ_0_ and δ_1_):

(6) $$CIV_{QALY}=\frac{\beta_{0}+\beta_{1}}{\delta_{0}+\delta_{1}}$$

The corresponding values represent the marginal rate of substitution between income and health with respect to well‐being, based on the overall population average. *CIV*_*QALY*_ thereby is the empirical conceptualisation of *v*_*Q*_ using the well‐being valuation approach. Income outliers (as will be defined in Section 3.4.1) were dropped from the baseline analysis.

### 3.3 Instrumental variable specification

A well‐documented problem of the well‐being valuation approach is the endogeneity of the income coefficient estimate. This was frequently addressed using an instrumental variable (IV) (see e.g., Brown, 2015; Howley, 2017; McNamee & Mendolia, 2018). Huang et al. (2018) instrumented income with the occurrence of financial‐worsening‐events such as personal bankruptcy or large financial losses.

Lacking such information, we followed Luechinger (2009), who used predicted labour‐market earnings based on industry‐occupation cells as income instrument. The rationale is that shifts in predicted income correspond to industry and/or occupation wide trends, which correlate with the development of negotiated wages or collective wage agreements, but do not reflect individual‐level effort or circumstances. Further, it is assumed that the income variance across industries and occupations captures information on the unobserved costs of income generation such as stress and/or associated health risks, and that unobserved selection effects of certain types of individuals into industries and occupations are captured in the time‐invariant fixed‐effects. One advantage of this instrument is that the captured income shifts have a rather permanent nature, whereas financial‐worsening‐events or lottery wins can be highly transitory shocks. In addition permanent income shifts have been found to be of higher relevance for individuals' well‐being (Bayer & Juessen, 2015; Cai & Park, 2016).

The identifying assumption is, therefore, that income variation across industries and occupations over time is uncorrelated with individual‐level characteristics and especially life satisfaction, besides the effect of income changes themselves. To implement the IV approach we followed a two‐stage least squares estimation procedure. In a first step we estimated the individual's labour market earnings *L*_*irt*_ based on the following regression;

(7) $$L_{irt}=\alpha +\rho_{0}I_{irt}+\rho_{1}O_{irt}+\rho_{2}T_{irt}+\rho_{3}R_{irt}+\mu_{r}+\epsilon_{t}+u_{irt}$$

from which we obtained fitted values, constituting the predicted labour earning conditional on the individual's industry‐occupation cell (*I*_*irt*_ and *O*_*irt*_), work tenure (*T*_*irt*_), and work‐hours (*R*_*irt*_) and a set of industry‐ and year‐fixed‐effects.

The obtained predicted labour earnings were summed on the household level and weighted by household composition to obtain the predicted household labour income ${\hat{L}}_{irt}^{HH}$, the instrument used in the first‐stage regression;

(8) $$Y_{irt}=\bar{\alpha }+{\bar{\beta }}_{0}H_{irt}+{\bar{\beta }}_{1}H_{irt-1}+{\bar{\delta }}_{0}{\hat{L}}_{irt}^{HH}+{\bar{\delta }}_{1}{\hat{L}}_{irt-1}^{HH}+\bar{\tau }X_{irt}+{\bar{\lambda }}_{i}+{\bar{\mu }}_{r}+{\bar{\epsilon }}_{t}+{\bar{u}}_{irt}$$

from which we obtained the fitted values for individual income, ${\hat{Y}}_{irt}$. In the second stage we substituted income *Y*_*irt*_ by ${\hat{Y}}_{irt}$, estimating

(9) $$W_{irt}=\alpha^{I}+\beta_{0}^{I}H_{irt}+\beta_{1}^{I}H_{irt-1}+\delta_{0}^{I}{\hat{Y}}_{irt}+\delta_{1}^{I}{\hat{Y}}_{irt-1}+\tau^{I}X_{irt}+\lambda_{i}^{I}+\mu_{r}^{I}+\epsilon_{t}^{I}+u_{irt}^{I}.$$

The resulting coefficients for health ($\beta_{0}^{I}$ and $\beta_{1}^{I}$) and income ($\delta_{0}^{I}$ and $\delta_{1}^{I}$) were then included in Equation (6) to calculate the IV *CIV*_*QALY*_ estimate. For further details please see Supplementary Appendix A3.

### 3.4 Alternative model specifications

#### 3.4.1 Treatment of outliers

Due to a right‐skewed and long‐tailed income distribution, with self‐reported income often misreported or even exaggerated (Hariri & Lassen, 2017), income outliers may have a large effect on *CIV*_*QALY*_ estimates when using linear models (Rousseeuw & Leroy, 1987). To identify outliers, which remains challenging for fixed‐effects models (Verardi & Croux, 2009), we reformulated our base case model as a pooled OLS model and calculated DFbeta, a measure quantifying the impact that dropping an observation has on the coefficient estimate. All observations with a DFbeta larger than 1, the recommended threshold (Bollen & Jackman, 1985), were dropped from the baseline analysis. In a robustness check we repeated the calculations including these outliers.

#### 3.4.2 Income specification

To accommodate the diminishing marginal return of income we log‐transformed income (Layard et al., 2008). *CIV*_*QALY*_ was then estimated based on a slightly modified equation as used by Ólafsdóttir et al. (2020) and van den Berg and Ferrer‐i‐Carbonell (2007). This entailed dropping the lagged income and health coefficients as used in our base model (Equation 6).

(10) $$CIV_{QALY}=\bar{y}*\left(exp\left(\frac{-\beta_{0}*\frac{1}{\Delta }}{\delta_{0}}\right)-1\right)*\Delta$$

In the log‐income specification *CIV*_*QALY*_ was calculated as the percentage share of annual income (median annual income $\bar{y}$). By construction, *CIV*_*QALY*_ values would be confined to be no greater than this income level which may be acceptable when valuing small gains or changes but not a full QALY. Therefore, we added the parameter Δ to the equation and set it to 10. Instead of calculating the monetary equivalent of a one QALY change we calculated the equivalent of a 0.1 QALY change and multiplied it by 10.

To account for the non‐linearity of income without imposing a logarithmic functional form, which may not adequately capture the relationship especially on the lower end of the income distribution, we furthermore tested a piecewise linear specification similar to Ólafsdóttir et al. (2020). To obtain the appropriate number of income splines and cut‐off values, we iteratively combined income‐deciles. The equality of coefficient estimates of adjacent splines was tested and non‐significantly different splines were gradually combined until coefficients were significantly different and model fit did not improve. *CIV*_*QALY*_ values were then calculated for each income spline and also aggregated by weighting according to the number of individuals in the respective splines. Estimating a piecewise IV specification was not feasible, as one distinct income instrument would have been required for each of the splines.

#### 3.4.3 Choice of utility tariff

Lacking a German specific SF‐6D utility tariff we relied on the UK time‐trade‐off based value set (Brazier & Roberts, 2004) to construct health utilities. In an alternative specification we explored the importance of tariff choice by instead applying a recently developed value set from the Netherlands which was estimated using a discrete choice experiment (Jonker et al., 2018).

#### 3.4.4 Health state dependence of the utility of consumption

Another empirical issue of concern relates to the interaction between health and income and experienced (consumption) utility. This so‐called health state dependence implies that the marginal utility gain from a given income change is directly dependent on the underlying health status (Finkelstein et al., 2013). So far, there is only inconclusive evidence on the magnitude and the direction of this effect: Finkelstein et al. (2013) found a negative health state dependence, a higher marginal utility of income in good compared to bad health, based on US data. However, replicating their approach using European data, Kools and Knoef (2019) found evidence for positive health state dependence, potentially due to differing provision of public goods in European healthcare systems.

As illustrated by both Finkelstein et al. (2013) and Kools and Knoef (2019), health state dependence has important implications for (health) economic issues such as the optimal design of insurance contracts or individual‐level decisions on life‐cycle savings. In the context of estimating *CIV*_*QALY*_, which requires a simultaneous measurement of the well‐being impacts of both health and income separately, a thorough investigation of the life‐cycle development of health states and the associated changes in consumption utility seems warranted.

To explore the potential impact of health state dependence on *CIV*_*QALY*_ estimates, we reduced our sample to those individuals that transitioned between health states. Finkelstein et al. (2013) used the onset of chronic diseases for this purpose. While this represents a convenient definition for an elderly population, we took a different approach, allowing us to observe the transition of individuals from good to bad health also for healthier groups. First, we reduced the sample to individuals whose mental or physical short form health questionnaire (SF‐12) component scores changed by at least 10, or one standard deviation, throughout their respective observation period.^3^ This was done to ensure that individuals in this group have experienced a consequential change in their mental and/or physical health. Good health states were defined as periods in which either of the two scores was above their respective individual‐level mean; bad health states if they were below. Secondly, we conditioned on the consecutive observation of differing health states with at least two consecutive periods needed to be observed in either state. This allowed us to estimate *CIV*_*QALY*_ for good and bad health separately while also ensuring that individuals transition into longer‐term health states (see Supplementary Appendix A4 for details). Importantly, the sample included individuals transitioning from good to bad health and vice versa, although the former is most frequent.

## 4 DATA

We used data from the annual SOEP panel survey, providing a representative sample of the adult (aged 16+) German population (Goebel et al., 2019). Ethical approval with respect to the surveying process generating the underlying data was obtained by the SOEP researchers directly. SF‐6D health utilities were constructed from SF‐12 data, which is biennially included in the survey since 2002. To facilitate the specified two‐year time‐frame *T* used for the *CIV*_*QALY*_ calculations, and to prevent dropping observations from every second year, we linearly imputed SF‐6D values for intermediate years. However, this was only done if individuals were observed for three consecutive years with two completed SF‐12 surveys.

Life satisfaction was measured on a 10‐point scale ranging from 0 (“completely dissatisfied”) to 10 (“completely satisfied”). Information on individuals' income was based on self‐reported monthly net household income. To account for differences in household composition, we calculated equivalized household income, following the definition by Hagenaars et al. (1994). Income data was converted to 2018 prices using the official consumer price indices (Federal Statistical Office, 2020).

To construct our instrument we extracted information on net labour income and individuals' industry and occupation. We dropped households with individuals where information on labour income but not on industry/occupation was available. Predicted labour income was assumed to be zero for all individuals with no labour income information, or who stated that they were not employed.^4^

We furthermore extracted information on a similar set of variables as used by Huang et al. (2018) to control for confounding factors. These included age, disability, marital status, employment status, educational attainment and leisure time. Table 1 summary statistics of the analysis data, consisting of 29,735 individuals providing 186,906 individual‐year observations. Supplementary Table A1.1 provides an overview of the conditioning applied to the SOEP data, while Supplementary Table A1.2 shows that the sub‐sample of employed individuals who were dropped because of missing industry/occupation information is comparable to the remaining sample of employed individuals. As the exclusion of individuals without at least two consecutive SF‐6D values was the only major selection criterion, the sample remained largely representative for the overall German population.

**TABLE 1:** Descriptive statistics

Variable	Mean	Std. Dev.	Description
Life satisfaction	7.09	1.71	0 (lowest) to 10 (highest)
Income in 1000's	2.03	1.29	Monthly household income in €
SF‐6D utility	0.73	0.13	0.345‐1, 1 perfect health
Disability	0.14	0.35	1 if disability status
Age in years	53.67	15.78	
(de facto) Married	0.67	0.47	1 if married, living together
Education: Primary	0.12	0.32	1 if primary educated
Education: Tertiary	0.63	0.48	1 if secondary educated
Education: Secondary	0.25	0.43	1 if tertiary educated
Leisure time	2.18	2.03	Hours per day
Employed	0.56	0.50	1 if employed
Unemployed	0.04	0.21	1 if unemployed
Work hours	21.22	20.99	Hours per week
Tenure	7.03	9.96	Years at current job
Individuals ∗ years		186,902	
Individuals		29,735	

Source: Own calculations based on SOEP Waves 2002–2018.

## 5 RESULTS

### 5.1 Baseline results

The baseline OLS and IV results, are shown in Table 2, separating between results using the full dataset with imputed SF‐6D values, and the dataset without imputation. To construct our instrumental variables, we predicted labour incomes based on industry/occupation for 125,229 observations. Supplementary Appendix A3 provides details on this prediction and the associated errors, which were small for the largest part of the income distribution. The instruments were significant in the first stage regression (Supplementary Table A3.1) and passed the Cragg‐Donald weak identification test (*F*‐value: 1864 and 192). This indicates a high relevance of the instrument, a common finding for this type of instrument (Bayer & Juessen, 2015; Luechinger, 2009). The Hausman test for endogeneity of the instrumented variables was significant, signalling that income should not be treated as exogenous.

**TABLE 2:** Baseline results

	SF‐6D imputation				No imputation			
	OLS		IV		OLS		IV	
Income in 1000's	0.05***	(0.01)	0.10***	(0.03)	0.05***	(0.01)	0.14***	(0.05)
Income in 1000's (*t* − 1)	0.01	(0.01)	0.04	(0.03)	−0.00	(0.01)	−0.00	(0.07)
SF‐6D utility	3.12***	(0.06)	3.12***	(0.05)	3.52***	(0.06)	3.51***	(0.05)
SF‐6D utility (*t* − 1)	0.10*	(0.06)	0.10*	(0.05)	0.47***	(0.05)	0.46***	(0.05)
Disability	−0.14***	(0.02)	−0.14***	(0.02)	−0.09***	(0.03)	−0.09***	(0.02)
Age	0.09***	(0.01)	0.08***	(0.02)	0.05***	(0.01)	0.05***	(0.01)
Age squared	−0.00***	(0.00)	−0.00**	(0.00)	−0.00**	(0.00)	−0.00	(0.00)
(de facto) Married	0.18***	(0.02)	0.18***	(0.02)	0.17***	(0.03)	0.16***	(0.02)
Primary education	−0.18*	(0.09)	−0.21***	(0.08)	−0.10	(0.15)	−0.13	(0.13)
Tertiary education	−0.18***	(0.06)	−0.19***	(0.05)	−0.19***	(0.07)	−0.20***	(0.07)
Leisure time	0.03***	(0.01)	0.03***	(0.00)	0.03***	(0.01)	0.03***	(0.01)
Leisure time squared	−0.00***	(0.00)	−0.00***	(0.00)	−0.00**	(0.00)	−0.00***	(0.00)
Unemployed	−0.52***	(0.03)	−0.53***	(0.02)	−0.53***	(0.04)	−0.53***	(0.03)
Work hours	0.00***	(0.00)	0.00***	(0.00)	0.00***	(0.00)	0.00	(0.00)
Tenure	−0.01***	(0.00)	−0.01***	(0.00)	−0.01***	(0.00)	−0.01***	(0.00)
Model statistics								
Cragg‐Donald			1864				192	
Anderson			3642				382	
Endogeneity test			10.0				5.8	
BIC	540,754.8		540,994.6		250,099.1		236,537.9	
Observations	186,902		186,902		93,450		85,433	
Individuals	29,735		29,735		29,735		21,718	
CIV‐QALY in €	58,533		22,717		80,522		28,130	

Source: Own calculations based on SOEP Waves 2002–2018.

Abbreviations: BIC, Bayesian information criteria; CIV‐QALY, compensating income variation of one QALY; IV, instrumental variable; OLS, ordinary least squares.

*p < 0.10, **p < 0.05, ***p < 0.01.

Equivalized monthly household income, health status (SF‐6D utility), and their lagged values were positive and significant predictors of life satisfaction in the OLS specification. This was also the case when instrumenting for income, except that the lagged income coefficient was insignificant. We observed a two‐fold increase in the income coefficients in the IV model (0.048 vs. 0.098), a similar magnitude to what has been observed in previous studies using the SOEP (Bayer & Juessen, 2015; Pischke, 2011). Interestingly, the difference is minimal compared to what was observed by Huang et al. (2018), who reported an IV coefficient which was 130 times larger than the OLS coefficient (0.080 and 0.0006). Applying the estimated income and SF‐6D coefficients to Equation (6) resulted in a *CIV*_*QALY*_ value of €58,533 in the OLS model and €22,717 when instrumenting for income. This value represents the average amount of additional income necessary to maintain the same level of life satisfaction if a hypothetical health change of one QALY is imposed.

Without SF‐6D imputation, reducing our sample to 85,433 observations across 21,718 individuals, the OLS results increased by a factor of 1.38 to €80,522 while the IV‐based value increased by a factor of 1.24 to €28,130. These differences were driven by larger SF‐6D and income coefficients compared to the baseline calculations, possibly resulting from increased within‐person variance as the distance between observations is two years instead of one. For the remainder of the results presented, we will be using the full dataset with imputed SF‐6D values to make use of the largest amount of information available.

Table 3 columns 2–3 contains estimates for East and West Germany separately, motivated by the persisting differences in life satisfaction and income levels (Frijters et al., 2004; Vatter, 2020). OLS‐based *CIV*_*QALY*_ estimates were €75,748 in the West and €28,548 in the East. The IV‐based estimate was also higher in the West compared to the East (€20,750 and €12,982), although the relative difference was lower (factor of 3.64 and 2.20). In both models, this difference was mainly driven by a considerably larger income coefficients in the East, likely due to the prevailing income differences between West and East; observed average monthly equivalized income was €2140 in the West and only €1652 in the East.

**TABLE 3:** Results by region and time‐period

	Baseline		East		West		w/o 2007–2009		2002–2006		2010–2018	
	OLS	IV	OLS	IV	OLS	IV	OLS	IV	OLS	IV	OLS	IV
Income in 1000's	0.05***	0.10***	0.13***	0.18**	0.04***	0.07**	0.05***	0.11***	0.06***	0.29***	0.04***	0.09*
	(0.01)	(0.03)	(0.02)	(0.08)	(0.01)	(0.04)	(0.01)	(0.04)	(0.01)	(0.09)	(0.01)	(0.05)
Income in 1000's (*t* − 1)	0.01	0.04	0.00	0.03	0.01	0.04	0.01*	0.05	−0.00	0.10	0.01	0.04
	(0.01)	(0.03)	(0.02)	(0.06)	(0.01)	(0.03)	(0.01)	(0.03)	(0.01)	(0.08)	(0.01)	(0.04)
SF‐6D utility	3.12***	3.12***	2.90***	2.90***	3.18***	3.17***	3.16***	3.15***	2.93***	2.92***	3.08***	3.08***
	(0.06)	(0.05)	(0.13)	(0.12)	(0.07)	(0.07)	(0.07)	(0.07)	(0.15)	(0.15)	(0.08)	(0.08)
SF‐6D utility (*t* − 1)	0.10*	0.10*	−0.12	−0.12	0.16**	0.16**	0.10	0.09	0.06	0.06	−0.07	−0.07
	(0.06)	(0.05)	(0.12)	(0.12)	(0.07)	(0.07)	(0.07)	(0.07)	(0.14)	(0.14)	(0.08)	(0.08)
Model statistics												
Cragg‐Donald		1863.7		323.9		680.2		783.4		181.2		494.3
Anderson		3642.0		544.4		1265.5		1429.5		328.8		907.3
Endogeneity test		10.0		1.5		5.8		9.7		8.2		2.7
BIC	540,755	540,995	127,072	127,092	412,723	412,877	431,238	431,487	129,869	130,432	276,374	276,464
Observations	186,902	186,902	43,447	43,447	143,361	143,361	151,461	151,461	48,678	48,678	101,048	101,048
CIV‐QALY in €	58,533	22,717	20,750	12,982	75,748	28,548	54,567	20,574	56,640	7,720	70,572	24,811

Source: Own calculations based on SOEP Waves 2002–2018.

Abbreviation: BIC, Bayesian information criteria; CIV‐QALY, compensating income variation of one QALY; IV, instrumental variable; OLS, ordinary least squares.

*p* < 0.10, **p < 0.05, ***p < 0.01.

We investigated the (undesired) impact of macro economic conditions on *CIV*_*QALY*_ estimates by excluding the years of the financial crisis and recession in Germany (2007–2009). As shown in Table 4 (columns 4–6), this had only a minor impact on the OLS and IV *CIV*_*QALY*_ values (€54,567 and €20,574). However, estimates based on the pre‐crisis time periods 2002–2006 (€56,640 and €7720) were substantially lower compared to estimates based on data from 2010–2018 (€70,572 and €24,811). This resulted from larger estimated effects of income in earlier periods, which may both be a result of a positive trend in incomes or a shift in population preferences and values over the last decades. Supplementary Table A2.1 provides further results on age and gender subgroups.

**TABLE 4:** Income specifications

	Baseline		With Outliers		Log income		Piecewise
	OLS	IV	OLS	IV	OLS	IV	OLS
Income in 1000's	0.05***	0.10***	0.03***	0.10***			
	(0.01)	(0.03)	(0.01)	(0.03)			
Income in 1000's (*t* − 1)	0.01	0.04	0.01***	0.04			
	(0.01)	(0.03)	(0.00)	(0.03)			
SF‐6D utility	3.12***	3.12***	3.12***	3.12***	3.18***	3.16***	3.18***
	(0.06)	(0.05)	(0.06)	(0.06)	(0.05)	(0.05)	(0.05)
SF‐6D utility (*t* − 1)	0.10*	0.10*	0.10*	0.10*			
	(0.06)	(0.05)	(0.06)	(0.06)			
Log income					0.24***	0.63***	
					(0.02)	(0.13)	
1st income spline							0.43***
							(0.05)
2nd income spline							0.27***
							(0.05)
3rd income spline							0.11***
							(0.02)
4th income spline							0.01
							(0.01)
Model statistics							
Cragg‐Donald		1863.7		825.8		1329.9	
Anderson		3642.0		1529.4		1278.2	
Endogeneity test		10.0		12.9		9.7	
BIC	540,755	540,995	540,801	541,306	540,506	541,501	540,448
Observations	186,902	186,902	186,906	186,906	186,902	186,902	186,902
CIV‐QALY in €	58,533	22,717	82,484	22,782	153,877	81,649	97,486
Without 4th spline							19,515

*Note:* Instrumental variable did not pass weak identification tests for piecewise income specification. CIVs for piecewise regression represents population‐weighted averages of all splines or the first three splines (€7,347, €11,686, €29,548 and €409,810).

Source: Own calculations based on SOEP Waves 2002–2018.

Abbreviations: BIC, Bayesian information criteria; CIV‐QALY, compensating income variation of one QALY​; IV, instrumental variable; OLS, ordinary least squares.

*p < 0.10, **p < 0.05, ***p < 0.01.

### 5.2 Specifications related to income

Re‐estimating our baseline models including four individual‐year observations which were flagged as outliers lead to a considerably lower income coefficient in the OLS model (Table 4 columns 3–4). This increased the *CIV*_*QALY*_ value to €82,484. The IV estimates were only minimally affected by this (€22,782). The outlier observations corresponded to two individuals from the same household, which reported a drop in monthly income from €142,534 to €14,051 within 2 consecutive years, while reporting constant life satisfaction.

In the models using log‐transformed income (Table 4 columns 5–6), the income coefficient was 0.24, larger than reported before by Pischke (2011) (0.125 to 0.182), who also used the SOEP. The corresponding IV coefficient, with a value of 0.63, was on the higher end of previous IV estimates based on the industry‐wage structure and the SOEP: Luechinger (2009) reported an estimate of 0.55, while Pischke (2011) reported values ranging from 0.489 to 0.617. Previous estimates based on instruments using lagged or future income shocks were also similar, with Bayer and Juessen (2015) providing a range of 0.45 to 0.50 for permanent income shifts.^5^ The log‐transformation resulted in considerably larger *CIV*_*QALY*_ values compared to the baseline. The OLS values increased by a factor of 2.63 to €153,877 while the IV values increase by a factor of 3.59 to €81,649.^6^

**FIGURE 1:**
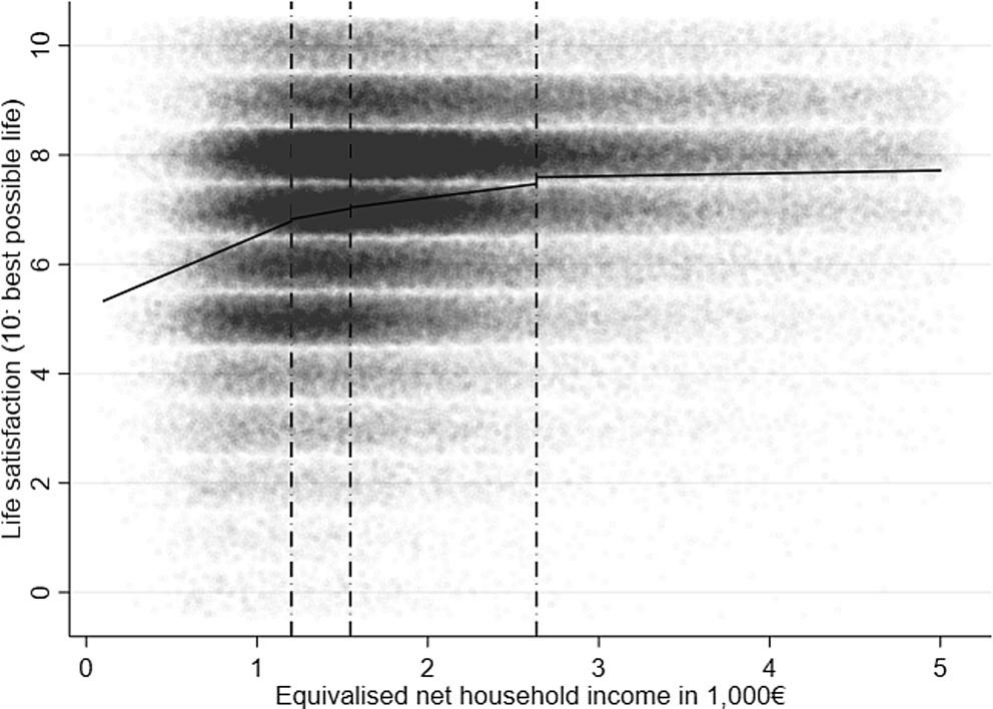
Relationship between life satisfaction and income across income splines. Life satisfaction values are depicted as small grey dots. Black dash‐dotted vertical lines represent the income splines used in the piece‐wise linear regression. Black horizontal lines plot the linear t within these splines

The piecewise linear specification was estimated with ultimately four income splines. The cut‐off points were at the 20^*th*^ percentile (€1200), the 40^*th*^ percentile (€1546), and the 80^*th*^ percentile (€2635). Figure 1 plots the overall distribution of life satisfaction across income, and the linear fit of life satisfaction across splines, indicating a non‐linear, diminishing pattern. The spline‐specific *CIV*_*QALY*_ values were €7347, €11,686, €29,548 and €409,810. The population aggregated *CIV*_*QALY*_ was €97,486. This estimate was driven by the large *CIV*_*QALY*_ value in the fourth income spline, where the income coefficient was insignificant. Using the three significant splines lead to a *CIV*_*QALY*_ value of €19,515.

### 5.3 Specifications and issues related to health

Choice of SF‐6D value set.

**FIGURE 2:**
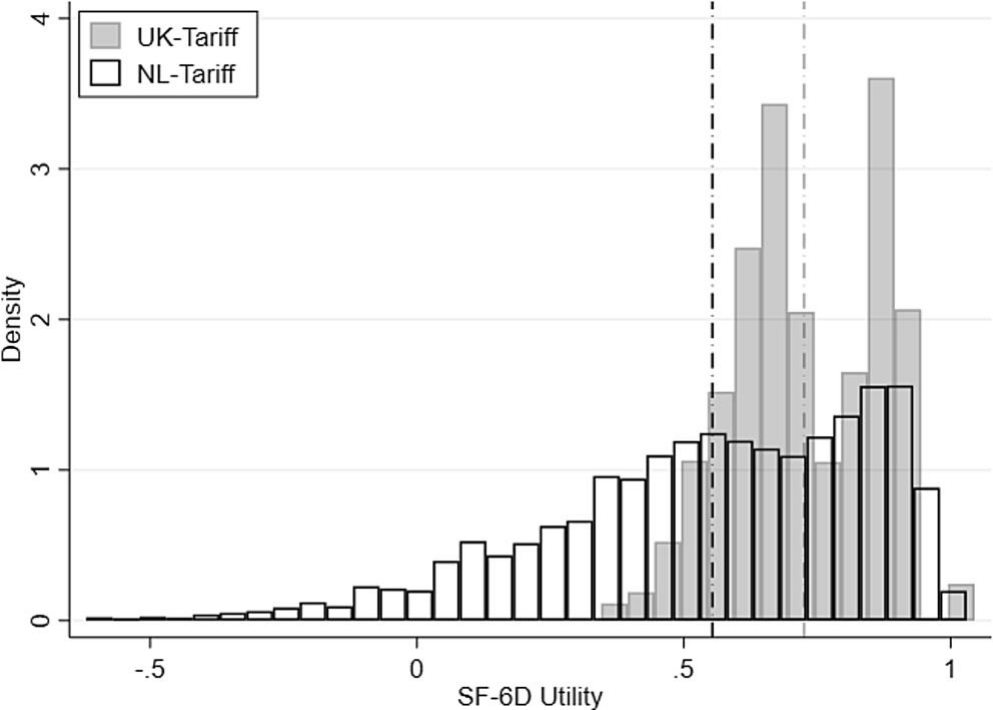
SF12 index values using UK and Dutch tariffs. The black dash‐dotted line indicates the Dutch tari mean. The grey dash‐dotted line indicates the UK tari mean. The distributions and means reect SF‐6D values based on self‐reported SF12 questionnaires only

Applying the Dutch SF‐6D value set shifted the distribution of health utilities (Figure 2), with the mean utility decreasing from 0.725 to 0.554. These differences likely reflect methodological differences rather than actual variation in health state preferences between the UK and the Netherlands as UK and Dutch tariffs for the EQ‐5D have been shown to be similar (Norman et al., 2009).

The estimated *CIV*_*QALY*_ values using the Dutch SF‐6D tariff were markedly smaller (Table 5). The OLS estimates decreased from €58,533 to €32,534, while the IV estimates decreased from €22,717 to €13,054. This shift was caused by the smaller SF‐6D coefficients (3.12 to 1.78), resulting from the wider spread of the Dutch tariff, which ranges from −0.44 to 1, allowing for negative health state utility, instead of 0.345 to 1 as in the UK value set. The same actual change in health corresponds to a larger change in SF‐6D utility in the Dutch tariff which reduces the impact of a (hypothetical) one unit change in SF‐6D on life satisfaction.

**TABLE 5:** Choice of SF‐6D tariffs

	UK Tariff		Dutch Tariff	
	OLS	IV	OLS	IV
Income in 1000's	0.05***	0.10***	0.05***	0.09***
	(0.01)	(0.03)	(0.01)	(0.03)
Income in 1000's (*t* − 1)	0.01	0.04	0.01	0.05*
	(0.01)	(0.03)	(0.01)	(0.03)
SF‐6D utility	3.12***	3.12***	1.78***	1.78***
	(0.06)	(0.05)	(0.03)	(0.03)
SF‐6D utility (*t* − 1)	0.10*	0.10*	0.05	0.05
	(0.06)	(0.05)	(0.03)	(0.03)
Model statistics				
Cragg‐Donald		1863.7		907.1
Anderson		3642.0		1671.4
Endogeneity test		10.0		9.4
BIC	540,755	540,995	538,297	538,523
Observations	186,902	186,902	186,902	186,902
CIV‐QALY in €	58,533	22,717	32,534	13,054

Source: Own calculations based on SOEP Waves 2002–2018.

Abbreviations: BIC, Bayesian information criteria; CIV‐QALY, compensating income variation of one QALY​; IV, instrumental variable; OLS, ordinary least squares.

*p < 0.10, **p < 0.05, ***p < 0.01.

Health state dependence of the utility of consumption

We explored the potential impact of health state dependence on *CIV*_*QALY*_ estimates by restricting our sample to individuals experiencing a substantial health change, and splitting their respective observation periods into good and bad health states (see Section 3.4.4). The resulting sample was considerably smaller, including only 5112 individuals yielding 48,861 observations. Nevertheless, the summary statistics suggests that the sample is still comparable to the full population sample (see Supplementary Table A4.1). Table 6 depicts the corresponding estimation results. Compared to the baseline estimates using the full sample, *CIV*_*QALY*_ values based on the combined good and bad health state samples were lower in the OLS model (€39,482) and similar in the IV specification (€20,377). For “good health states”, the corresponding *CIV*_*QALY*_ estimates were lower with €33,336 and €16,532. For “bad health states”, the OLS‐based *CIV*_*QALY*_ estimate was €38,374 and the IV‐based estimate €11,779.Important to note is that the drop in the IV based results for the bad health state primarily resulted from a larger income coefficient estimate, even though the SF‐6D coefficients increased considerably. These results indicate that there is a positive health state dependence of income in line with the results for Germany by Kools and Knoef (2019). Unfortunately, we were not able to follow Kools and Knoef (2019) and Finkelstein et al. (2013) in focusing on non‐working individuals to ensure stable income across health states, ruling out that the increased income coefficients are driven by individuals losing their income, and hence having a larger marginal utility of additional earnings. For our analysis, such a restriction was not feasible, as within‐person income variation is necessary to estimate the income coefficients. However, the general empirical pattern remains the same when excluding individuals with large negative income differences between health states (see Supplementary Table A4.2). This also holds when only considering the working population (Supplementary Table A4.3) and those experiencing sudden and severe health changes (Supplementary Table A4.4).

**TABLE 6:** Health state dependence

	Baseline		Good health		Bad health	
	OLS	IV	OLS	IV	OLS	IV
Income in 1000's	0.07***	0.17**	0.05***	0.11	0.08**	0.32
	(0.01)	(0.07)	(0.02)	(0.08)	(0.04)	(0.24)
Income in 1000's (*t* − 1)	0.03**	0.02	0.03**	0.05	0.03	0.05
	(0.01)	(0.06)	(0.01)	(0.06)	(0.03)	(0.17)
SF‐6D utility	3.62***	3.60***	2.51***	2.50***	4.10***	4.03***
	(0.11)	(0.09)	(0.14)	(0.12)	(0.38)	(0.37)
SF‐6D utility (*t* − 1)	0.10	0.11	0.12	0.12	0.32	0.32
	(0.10)	(0.10)	(0.12)	(0.11)	(0.26)	(0.27)
Model statistics						
Cragg‐Donald		620.7		425.1		95.9
Anderson		1208.4		828.1		188.4
Endogeneity test		3.0		1.8		1.0
BIC	150,481	150,558	102,463	102,497	37,832	37,899
Observations	48,861	48,861	35,401	35,401	13,460	13,460
CIV‐QALY in €	39,482	20,377	33,336	16,532	38,374	11,779

Source: Own calculations based on SOEP Waves 2002–2018.

Abbreviations: BIC, Bayesian information criteria; CIV‐QALY, compensating income variation of one QALY​; IV, instrumental variable; OLS, ordinary least squares.

*p < 0.10, **p < 0.05, ***p < 0.01.

### 5.4 Robustness checks

Lastly, we tested the robustness of our baseline results to some general concerns regarding our estimation strategy (Table 7). In a first robustness check, we limited our sample to individuals which were in paid employment and provided industry‐occupation information, the same sample which was used to obtain estimates for predicted labour income for the IV regression. The resulting OLS‐based *CIV*_*QALY*_ was slightly lower than the baseline at €52,829, while the IV‐based value was slightly higher than the baseline at €26,097. These differences were driven by the smaller SF‐6D coefficients in both OLS and IV models, likely resulting from the working population being healthier as individuals without labour income (the unemployed and retired). The sum of both income coefficients was smaller in the corresponding IV‐calculations compared to baseline, increasing the *CIV*_*QALY*_.

**TABLE 7:** Robustness checks

	Baseline		Working only		No self‐employed		No bonus income		With ind/occ missing
	OLS	IV	OLS	IV	OLS	IV	OLS	IV	OLS
Income in 1000's	0.05***	0.10***	0.05***	0.05	0.07***	0.05	0.05***	0.14***	0.04***
	(0.01)	(0.03)	(0.01)	(0.03)	(0.01)	(0.04)	(0.01)	(0.04)	(0.01)
Income in 1000's (t − 1)	0.01	0.04	0.01	0.07**	0.00	0.08**	0.01*	0.02	0.01**
	(0.01)	(0.03)	(0.01)	(0.03)	(0.01)	(0.03)	(0.01)	(0.03)	(0.00)
SF‐6D utility	3.12***	3.12***	2.95***	2.94***	2.97***	2.97***	3.12***	3.11***	3.14***
	(0.06)	(0.05)	(0.08)	(0.07)	(0.08)	(0.07)	(0.07)	(0.06)	(0.06)
SF‐6D utility (t − 1)	0.10*	0.10*	0.07	0.06	0.01	0.01	0.11*	0.11*	0.12**
	(0.06)	(0.05)	(0.07)	(0.07)	(0.08)	(0.07)	(0.06)	(0.06)	(0.06)
Model statistics									
Cragg‐Donald		1863.7		1355.7		1898.4		719.1	
Anderson		3642.0		2637.7		3633.0		1334.4	
Endogeneity test		10.0		5.4		7.4		10.1	
BIC	540,755	540,995	319,169	319,323	279,896	280,043	502,827	503,172	578,002
Observations	186,902	186,902	116,125	116,125	101,703	101,703	172,998	172,998	198,950
CIV‐QALY in €	58,533	22,717	52,829	26,097	44,058	21,382	53,974	20,464	62,266

*Note:* Ind/occ refers to specification where individuals without industry/occupation information were included.

Source: Own calculations based on SOEP Waves 2002–2018.

Abbreviations: BIC, Bayesian information criteria; CIV‐QALY, compensating income variation of one QALY​; IV, instrumental variable; OLS, ordinary least squares.

*p < 0.10, **p < 0.05, ***p < 0.01.

Next, we followed Luechinger (2009) by excluding households with self‐employed main income earners, as the income measurement error was likely to be amplified among these individuals. Self‐employed individuals are often reluctant to disclose their income, while also experiencing unstable income streams and hence, even if not reluctant to report, they might simply misreport accidentally. The resulting *CIV*_*QALY*_ estimates and income and SF‐6D coefficients were similar to the baseline estimates (€55,359 and €20,352).

Another concern relating to the instrument is that observed income changes may also relate to individual effort, which likely impacts income differently across industries and occupations. Unfortunately, effort cannot be observed. To nevertheless explore this, we use information on reported bonuses, gratifications, or profit sharing to identify the group of individuals for whom this might be a relevant concern, as for them effort would have the highest impact on income and life satisfaction. To test the robustness of our results to this potential bias, we estimate our baseline models excluding such observations. The results in Table 7 columns 7–8 suggest that this bias is relatively limited.

To investigate the potential impact of dropping employed individuals without industry/occupation information (as required for constructing the IV), we included those observations in a further robustness check (Table 7 column 9). The corresponding OLS estimates for income coefficients and *CIV*_*QALY*_ (€62,266) are comparable to our baseline estimates. However, by construction, we cannot confirm this for the IV estimates.

## 6 DISCUSSION

Applying the well‐being valuation approach to longitudinal health and income data from Germany, we estimated the monetary equivalent value of one year in full health *v*_*Q*_ (equivalent to one QALY). Beyond demonstrating the feasibility of this approach in a new country context, we explored additional empirical and methodological challenges with implications for the practical usefulness of well‐being valuation based *v*_*Q*_ estimates (denoted as *CIV*_*QALY*_).

### 6.1 Overview and context of results

**FIGURE 3:**
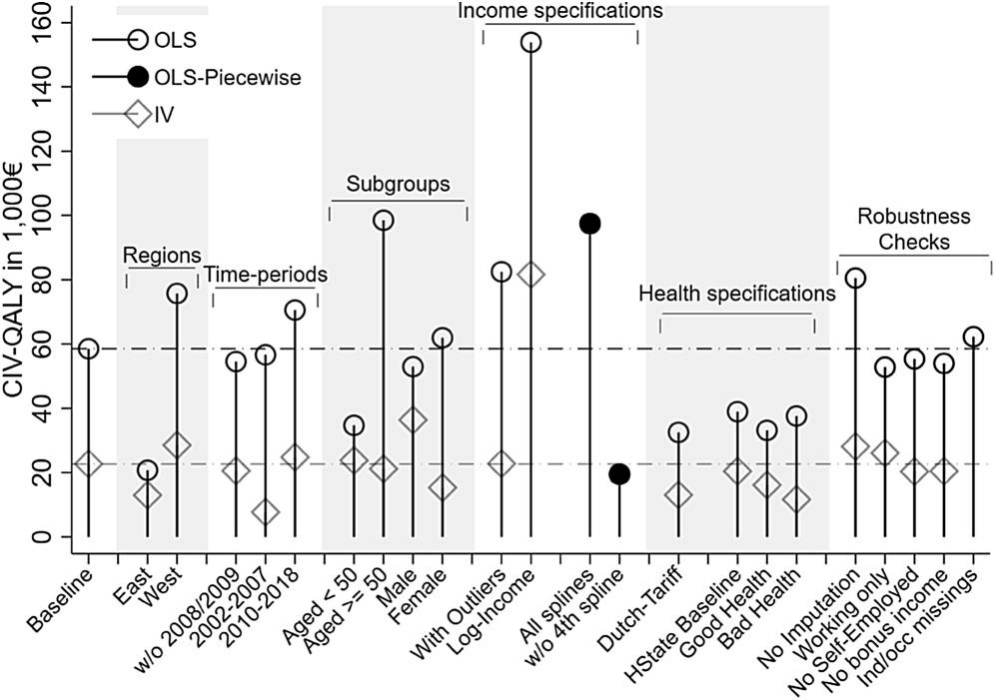
Overview of *CIV*_*QALY*_ estimates. The horizontal dash‐dotted lines indicate our baseline CIVQALY estimates from the baseline OLS (black) and IV (grey) specications

Figure 3 presents an overview of our *CIV*_*QALY*_ estimates. The baseline calculations provided average monetary valuations of a QALY of €58,533 (OLS) and €22,717 (IV). *CIV*_*QALY*_ estimates varied across model specifications with the bulk of values lying between €20,000 and €60,000 and the (OLS) log‐income specifications reaching the maximum value of €153,877. Instrumenting for income consistently lead to lower values, a common finding in the well‐being valuation literature (e.g., Ólafsdóttir et al. (2020)).

The range of *CIV*_*QALY*_ estimates obtained in our study fit into the ballpark of more reasonable stated preference estimates (Ryen & Svensson, 2015). Furthermore, it is important to note that all IV *CIV*_*QALY*_ estimates, except the log‐income specification, fell within the range of *v*_*Q*_ estimates for Germany of €4988 to €43,115 reported by Ahlert et al. (2016), who provided the only *v*_*Q*_ estimates until now. A first approximation of an *opportunity cost based QALY threshold value*, or *k*_*Q*_, for Germany was reported by Woods et al. (2016). Using empirical estimates of health care opportunity costs for Germany, and the relationship between GDP per capita and the value of a statistical life, they calculated a *k*_*Q*_ range of €19,276 to €24,374 (in 2018 euros). A recent related study by Ochalek and Lomas (2020) reported estimates of cost per DALY averted (essentially the reciprocal of a QALY gain) for Germany of €47,116 to €74,650 (in 2018 euros).

### 6.2 Limitations and strengths of the analysis

IV‐based estimates rely on restrictive assumptions relating to their unbiasedness and informational value. A valid concern is that occupational choice may be related to other unobserved confounders, such as personality traits or income preferences (Pischke & Schwandt, 2012). The use of individual fixed‐effects should somewhat alleviate such concerns due to the rather stable nature of personality traits (Borghans, Duckworth, Heckman, & ter Weel, 2008), but they cannot provide complete assurance. A further assumption is that being employed in a certain industry/occupation should not have a significant, direct effect on life satisfaction, therefore violating the exclusion restriction. Supplementary Figures A3.6 and A3.7 show that, controlling for income and other confounders, this effect is not zero, but modest and mostly insignificant. One additional drawback that is rarely explicitly discussed but of great importance in the well‐being valuation context, is that IV estimates only yield a local average treatment effect (Angrist et al., 1996). Using predicted labour income as an instrument, at least questions the generalisability of our IV estimates to the full, also non‐working, population. Further, as we are not able to address all sources of measurement error with respect to income, the remaining upward bias in the income coefficients would imply a downward bias in the estimated *CIV*_*QALY*_ values.

In addition, income variation in industry‐occupation cells predominantly consists of *positive*, *upward shifts in wages* (and differences therein). This is conceptually different to financial worsening events, as used by Huang et al. (2018), as these capture *income losses*.^7^ Given income loss aversion (Boyce et al., 2013), our IV based *CIV*_*QALY*_ estimates likely represent a lower‐bound.

The potential endogeneity of health (status) in life satisfaction regressions due to reverse causality (see e.g., Veenhoven, 2008 or Sabatini, 2014), which is rarely addressed in the related literature, is a further limitation. This endogeneity could be addressed by appropriate instruments or identifying health shocks which are plausibly exogenous, such as heart attacks or strokes. However, besides practical issues like data availability, it is questionable how generalisable such localized causal effects would be for the overall impact of the multi‐dimensional construct of health on life satisfaction. Heterogeneity may exist both concerning the type of health shocks, but also relating to their timing within the (life‐cycle) health distribution. Whether or not our estimates of the impact of health are biased upwards or downwards can therefore not be easily ascertained. In the one previous article in the related literature that addressed endogeneity directly, Brown (2015) found that the health coefficient was slightly overestimated when not instrumented. Assuming this also holds in our context, this would imply that there is an upward bias in our *CIV*_*QALY*_ values resulting from the endogeneity of health.

A more practical limitation relating to measuring health was that we had to impute SF‐6D utilities for every second year to make full use of the SOEP's rich annual data. This required us to condition the sample on individuals who had at least three consecutive observations, which may have resulted in underestimating the impact of deteriorating health, since individuals are more likely to discontinue their participation in a longitudinal survey following a negative health shock.

A final limitation lies in the potential presence of double‐counting as SWB enters the model twice: As an implicit consideration in the SF‐6D health state valuation tasks (on which the scoring of our health measure is based on), and as a proxy for experienced utility (Equation 2). To what extent this is problematic is difficult to assess. To avoid this double counting one could use an unweighted sum score of the SF‐6D levels. However, this raises the question of the appropriate anchoring. Using such a sum score, rescaled to a 0 to 1 range (expanding the number of levels of the first two SF‐6D dimensions to five to not impose any weighting) lead to lower *CIV*_*QALY*_ estimates in the unimputed dataset (Supplementary Table A2.2, columns 4–5). However, when imposing the same anchor and therefore range as in the original SF‐6D tariff (0.345 to 1), the OLS and IV results (€88,867 and €30,567) were much closer to the unimputed baseline estimates (€80,671 and €27,777).

It seems that not the differential weighting between the dimensions caused the larger differences, but the different anchors, that is the lowest utility. Another alternative approach entailed eliciting *CIV* values for different dimensions directly by regressing on all levels of the SF‐6D, which did not impose any weighting. Adding up the resulting *CIV* values of the lowest level of all six dimensions, summed up to a cumulative value of moving from the best possible to the worst possible health state of €79,013 and €27,489, which again resembled the unimputed baseline estimate (Supplementary Table A2.2). While these sensitivity checks somewhat alleviate the concerns about double‐counting, the latter revealed that 46% of the *CIV*_*QALY*_ value stemmed from the impact of mental health on life satisfaction. It is likely that the mental health dimension also plays a dominant role in our baseline calculations. Whether this in itself is problematic lies outside the scope of this paper, as it relates to a more general issue of the well‐being valuation approach: is life satisfaction the best (available) proxy for experienced utility?

### 6.3 Implications of findings

There are several practical implications of our study for future applications of the well‐being valuation approach in general, and its use for estimating *v*_*Q*_ in particular. First, judging from the impact outliers have in the OLS specification (Table 4), subsequent applications of the approach using linear models should report on the occurrence and treatment of outliers. Secondly, given that the functional form of income had a large impact on our estimates its final specification has to be well argued and reporting results for other alternative functional forms seems warranted. The piecewise linear specification seems to be a promising alternative, given that it is more flexible and gives all income groups a proportional weight. This approach, however, comes at the price of increasing the number of variables that need to be instrumented for.

Third, the choice of utility tariffs for the health instrument matters greatly. Especially the range of the scoring algorithm has a large impact (Supplementary Table A2.2), as an imposed one unit change in health utility implies a different change in health if the range goes from 0.345 to 1 or −0.44 to 1. How to overcome this issue while facilitating cross‐country comparisons and how this relates to the underlying QALY concept, should further be discussed in future applications. Lacking country specific tariffs, it may be convenient to opt for a tariff whose origin can be placed in cultural and socio‐economic proximity to the country to be investigated. However, the impact of methodological peculiarities in how these tariffs were generated is relevant. It would have been interesting also to compute *CIV*_*QALY*_ estimates based on the more widely used EQ‐5D health utilities and compare the implications of differences in scope and range of the health instrument used on *CIV*_*QALY*_ values. Unfortunately, EQ‐5D is rarely included longitudinal surveys. Lastly, the differing values obtained when considering East and West Germany separately, or specific time periods (Table 3), also highlight the potential importance of the specific country context for *CIV*_*QALY*_ calculations.

One of the major conceptual issues discussed in our analysis, with direct relevance for the practical value of any empirically estimated *CIV* of health, is the health state dependence of utility. We attempted to provide indicative evidence on how health state dependence might affect estimated *CIV*_*QALY*_ values. However, it remains unclear whether empirical approaches based on self‐reported (panel) data can produce reliable estimates if health state dependence is prevalent and survey participation and attrition is (partially) driven by health changes over time. We found considerable differences in the estimated *CIV*_*QALY*_ values when comparing periods of good and bad health within individuals (Table 6). As the underlying point estimates depicted substantial uncertainty, these findings should be interpreted with caution and merely as indicative evidence for the role of health state dependency in this context. The impact of this sub‐sample of individuals on the population wide *CIV*_*QALY*_ value is likely small, as attrition is high once individuals experience bad health states, long‐term or very severe health shocks. Hence, a pragmatist might argue that this issue is of theoretical interest only. We would argue, however, that this is an inherent limitation of self‐reported observational data and its *ex‐post* perspective in this context. Stated preference methods would allow for an explicit *ex‐ante* consideration of this issue through tailored sampling strategies and survey design.

An additional conceptual concern related to health state dependence is the question of adaptation to bad health over time (Huang et al., 2018). Adaptation implies the gradual return of SWB to pre‐health‐shock levels despite continued (or deteriorating) bad health (Loewenstein & Ubel, 2008). This phenomenon has been documented before using the SOEP‐data (Oswald & Powdthavee, 2008) and would generally decrease estimated *CIV*_*QALY*_, as the marginal utility of health would decrease with time spent in bad health. To what extend this represents an estimation error, however, is debatable and depends on what is perceived to be the “true” impact of ill‐health on well‐being over time, and whether adaptation, if present, should be corrected for. The recent findings by Etilé et al. (2020), who documented a heterogeneous distribution of adaptive potential across subgroups, underline the relevance of this concern also from a normative perspective.

The previous remarks highlight avenues for future research, like investigating the causal effect of health on life satisfaction, for example using instrumental variable regressions. In addition, the approach would crucially benefit from further research into the impact of income on life satisfaction, for example using (natural) experiments. The regular inclusion of variables that represent valid instruments for income into different population panel surveys could also be beneficial for further exploring the reliability and validity of these instruments and the approach as a whole, as it would allow cross‐national replications of results. Meanwhile, future applications may draw upon recent advances into the generalisability of IV‐based estimates (see e.g., Mogstad et al. (2018)) to explore how these concerns can be addressed within the framework of available instruments. Further, linking survey data on individual‐level SWB measures with detailed administrative records on income, health, and care consumption would also be a fruitful direction for further inquiry, resolving some of the enumerated concerns. With respect to the question of health state dependency, for example, it would be possible to determine the extent to which survey data has an inherent blind spot due to the attrition of individual following severe health shocks. In addition, such data could also be used to explore a wider range of specification choices within the general empirical strategy used, for example with respect to the choice of control variables. Here, we deliberately followed Huang et al. (2018), as the set of basic control variables they propose is available in most national panel surveys, which facilitates replications across country‐contexts. However, there is ample room for extending the analysis by considering a wider set of control variables and their impact on *CIV*_*QALY*_ estimates, or even to altogether choose a different approach such as shrinkage estimators (e.g., LASSO) or matching to address endogeneity concerns around the impact of health and/or income on life satisfaction.

A final issue concerns the practical application of our *v*_*Q*_ estimates. If certain (health) policies/interventions in Germany were to be evaluated using a *v*_*Q*_ value from our study, which range from around €20,000 (IV) to €60,000 (OLS), we have to highlight the following:^8^ Our study cannot provide a definite answer regarding which estimate is most accurate to be used in different contexts. This relates to the uncertainty surrounding these estimates and the underlying assumptions, but also to normative or distributional questions, which need to be addressed in the future (Cookson et al., 2020). While our piecewise regression results somewhat reflect such concerns by constructing *v*_*Q*_ estimates using a weighted mean of the different parts of the income distribution, this is only a first, very simplistic approach. When used in a normative context, like decisions on reimbursement of technologies, explicit policy (debate and) support is required. Applied studies could use the range we provided to highlight the impact of varying *v*_*Q*_ estimates on their results and recommendations, keeping in mind that for specific sub‐populations our *v*_*Q*_ estimates might not be directly applicable. In any case the selection of any specific value over another in any practical application should be transparently discussed with respect to the applied selection criteria.

## 7 CONCLUSIONS

We demonstrated that the well‐being valuation approach *can* be another useful instrument in the (health) economist's tool box for obtaining monetary equivalent valuations of health (*v*_*Q*_). Some inherent empirical and conceptual challenges of applying this approach in this context can be addressed, especially when using large‐scale longitudinal data. However, other issues, like the health state dependence of the utility of consumption, will remain a threat to the validity of estimates, warranting additional research. Concurrently, alternative approaches of estimating *v*_*Q*_, like stated preference studies or methods aiming at eliciting the value of a statistical life, as recently applied by Herrera‐Araujo et al. (2020), provide important complementary insights, despite their conceptual differences. Also given their respective strengths and limitations, methodological diversity is desired in the ongoing endeavour of measuring the monetary equivalent value of health.

The type of *v*_*Q*_ estimates provided in our analysis reflect average marginal health valuations (with the caveat of being entirely based on marginal changes in health related quality of life), representative on a national level. As such, these can be applied in economic evaluations informing decision making on a societal level for publicly funded policies or interventions. Such *v*_*Q*_ estimates predominantly find their use by informing the cost‐effectiveness threshold in the context of cost‐utility analysis within health care, which aid in informing decisions on reimbursement of certain health interventions. However, estimates of the monetary value of health can also be useful in broader contexts, like cost‐benefit analyses or similar approaches (Cookson et al., 2020), especially when benefits and costs of policies/interventions constitute a mix of health and non‐health outcomes occurring across different sectors. Advancing methodologies aiming to estimate *v*_*Q*_ and providing insights into their validity can assist in informing some of the uncomfortable trade‐offs that societies generally face in priority‐setting both within health care but also beyond (Chilton et al., 2020).

## CONFLICTS OF INTERESTS

None of the authors has any conflict of interest to declare.

## Supporting information

Supplementary Material

## Data Availability

The SOEP scientific use file with anonymized microdata is made available at no cost to universities and research institutes for non‐commercial research and teaching purposes in various data formats. To obtain data access users must sign a user contract and confidentiality agreement (http://www.diw.de/soep-contractmanagement). After approval of this contract the data can be downloaded from the SOEP website via a secure data transfer system. For further instructions please see the data access information on the SOEP website (https://www.diw.de/en/diw_01.c.601584.en/data_access.html). This publication used the SOEP core v35 (1984 ‐ 2018) data release. A replication package can be found on the Open Science Framework (https://osf.io/b8nsz/). This replication package includes all Stata dofiles used to obtain the presented results as well as those needed to construct the dataset used for the analyses based on the raw SOEP v35 data release.

## ENDNOTES

^1^
In the broader cost‐benefit framework, this quality‐adjusted life year (QALY) equivalent *v*_*Q*_ value can be used for transforming health gains into monetary benefits.

^2^
Instead, the trade‐off between Δ*c*_*t*_ and Δ*Q* is discussed and determined in closed‐door price negotiations between health authorities and the manufacturer. The methodological uncertainty around estimating *v*_*Q*_ has been cited as a key reason for the scepticism towards adopting more transparent threshold‐based decision rules (Bundesministerium für Gesundheit, 2008).

^3^
The SF‐12 is also used to calculate SF‐6D health utilities. Component scores range from 0 (worst) to 100 (best) with a normalized mean of 50 and standard deviation of 10 (Ware et al., 1995).

^4^
Following Luechinger (2009) we added a constant of €1 to all incomes for the log‐income specification.

^5^
Bayer and Juessen (2015) used only data from West Germany, possibly leading to a downward bias due to higher income levels in the West. Similarly, both Pischke (2011) and Luechinger (2009) use SOEP waves from the years before the East German SOEP sample was established in 1990 alongside waves containing samples from both former German states past 1990.

^6^
Huang et al. (2018) did not observe a large difference between linear and log income based estimates. However, they multiplied the ratio of income and health coefficients as in Equation (6) with the median income to obtain *CIV*_*QALY*_ (as opposed to Equation (10)).

^7^
Ambrosio et al. (2018) report a persistent direct effect of financial worsening (and improvement) events on life satisfaction beyond income‐changes, raising concerns on the general appropriateness such events as income instruments.

^8^
Health care funding decisions in Germany are not based on cost utility analysis, partially because thresholds were considered to be difficult to define (Bundesministerium für Gesundheit, 2008). Finding comparable monetary estimates using a compensating income variation and stated preference studies to some extent puts this into perspective.
