# Supplementary material for: The value of health—Empirical issues when estimating the monetary value of a quality‐adjusted life year based on well‐being data
Source: Health Econ. 2021 May 5;30(8):1849–70. doi: 10.1002/hec.4279 (PMC8360130; doi:10.1002/hec.4279)
Supplement: Supplementary file 1 — Supplementary Material [file HEC-30-1849-s001.pdf]

A1 Dataset Details

Table A1.1: Dataset conditioning

**Description**	**Observations**
Total number of individual-year observations (2002-2018)	434,002
Clean Control variables: sex, age, disability, marital/employment status	326,717
Remove working individuals without industry-occupation information	309,253
At least two consecutively observed self-reported SF12	243,157
Remove observation not included in consecutive triplets	220,358
Excluding observations without lag of SF12, income and disability	186,902
Observations	186,902
Individuals	29,735
**Without SF-6D-Imputation**	
Observations	85,433
Individuals	21,718

Source: Own calculations based on SOEP Waves 2002-2018. *Note:* The difference in the number of unique individuals between imputed and unimputed dataset occurs as we include current and lagged health state variables. For the unimputed dataset this requires a full set of three consecutive SF-6D values or the participation in at least five SOEP waves to yield the minimum number of two individual-year observations with full information that are needed for the fixed-effects specification.

Table A1.2: Characteristics of employed with and without industry/occupation information

**Variable**	**No ind/occ information**	**Information available**	**Description**
Life satisfaction	7.32 (1.61)	7.24 (1.56)	0 (lowest) to 10 (highest)
Income in 1000's	2.18 (1.53)	2.27 (1.39)	Monthly household income in €
SF-6D utility	0.75 (0.12)	0.75 (0.12)	0.345-1, 1 perfect health
Disability	0.05 (0.22)	0.07 (0.25)	1 if disability status
Age	42.95 (11.24)	45.68 (10.3)	
(de facto) Married	0.68 (0.47)	0.66 (0.47)	1 if married, living together
Primary education	0.1 (0.3)	0.07 (0.26)	1 if primary educated
edu_secondary	0.6 (0.49)	0.63 (0.48)	1 if secondary educated
Tertiary education	0.3 (0.46)	0.3 (0.46)	1 if tertiary educated
Leisure time	1.52 (1.33)	1.53 (1.31)	Hours per day
Work hours	35.98 (14.23)	37.73 (12.73)	Hours per week
Tenure	8.25 (9.56)	12.41 (10.4)	Years at current job
Observations	9,601	104,846	

Source: Own calculations based on SOEP Waves 2002-2018.

We constructed our dataset in a way that it can be used for both OLS and IV regressions on the same sample. Therefore we dropped individuals from the analysis, who were employed, but

did not provide information on industry/occupation, as this was needed for constructing the income instrument and setting their predicted labour income to zero would bias our estimates. Table A1.2 shows that there were no noteworthy differences in observable characteristics between employed individuals providing such information and those who did not. This alleviates concerns on inducing selection bias through this step of the data conditioning. The number of observations in the second column deviates from the number of observations used for predicting labour income (125,229) as dropping individuals without industry/occupation information was done at the end of the dataset conditioning, after several other steps (e.g. restricting to individuals with at least 3 observations).

A2 Appendix - Additional Results

Table A2.1: Subgroup results

	**Baseline**		**Age<50**		**Age≥50**		**Male**		**Female**	
	OLS	IV	OLS	IV	OLS	IV	OLS	IV	OLS	IV
Income in 1000's	0.05***	0.10***	0.07***	-0.01	0.03***	0.21***	0.04***	0.02	0.05***	0.20***
	(0.01)	(0.03)	(0.01)	(0.05)	(0.01)	(0.05)	(0.01)	(0.04)	(0.01)	(0.05)
Income in 1000's (t-1)	0.01	0.04	0.02**	0.14***	0.00	-0.06	0.01*	0.06*	0.00	0.02
	(0.01)	(0.03)	(0.01)	(0.04)	(0.01)	(0.04)	(0.01)	(0.04)	(0.01)	(0.04)
SF-6D utility	3.12***	3.12***	2.99***	3.00***	3.17***	3.16***	3.01***	3.01***	3.21***	3.21***
	(0.06)	(0.05)	(0.10)	(0.09)	(0.08)	(0.08)	(0.09)	(0.09)	(0.09)	(0.08)
SF-6D utility (t-1)	0.10*	0.10*	0.04	0.03	0.09	0.10	0.05	0.05	0.16*	0.14*
	(0.06)	(0.05)	(0.09)	(0.09)	(0.08)	(0.08)	(0.09)	(0.08)	(0.08)	(0.08)
**Model statistics**										
Cragg-Donald		1,863.7		661.1		265.5		513.5		398.7
Anderson		3,642.0		1,179.4		504.9		929.8		747.1
Endogeneity test		10.0		11.5		14.5		1.8		16.0
BIC	540,755	540,995	223,269	223,623	307,453	308,339	247,607	247,666	293,222	293,723
Observations	186,902	186,902	80,324	80,324	105,231	105,231	87,192	87,192	99,710	99,710
**CIV in €**	58,533	22,717	34,691	23,814	98,518	21,193	52,956	36,397	61,947	15,335

Source: Own calculations based on SOEP Waves 2002-2018. *Note:* * $p < 0.10$, ** $p < 0.05$, *** $p < 0.01$. BIC Bayesian information criteria.

Table A2.2: Results for unweighted and separate SF-6D levels

	**Baseline**				**SF-6D sum score**				**SF-6D levels**			
	OLS		IV		OLS		IV		OLS		IV	
Income in 1000's	0.05***	(0.01)	0.14***	(0.05)	0.05***	(0.01)	0.14***	(0.05)	0.05***	(0.01)	0.12**	(0.05)
Income in 1000's ($t-1$)	-0.00	(0.01)	-0.00	(0.07)	0.00	(0.01)	0.01	(0.07)	0.00	(0.00)	0.02	(0.07)
SF-6D utility	3.52***	(0.06)	3.51***	(0.05)								
SF-6D utility ($t-1$)	0.47***	(0.05)	0.46***	(0.05)								
SF-6D Summary Score					2.69***	(0.04)	2.69***	(0.04)				
SF-6D Summary Score ($t-1$)					0.30***	(0.04)	0.29***	(0.04)				
Physical Function 2									-0.05***	(0.01)	-0.06***	(0.01)
Physical Function 3									-0.19***	(0.02)	-0.19***	(0.02)
Role Function 2									-0.00	(0.01)	-0.00	(0.01)
Role Function 3									-0.18***	(0.02)	-0.18***	(0.02)
Role Function 4									-0.15***	(0.02)	-0.15***	(0.02)
Social Function 2									-0.07***	(0.01)	-0.07***	(0.01)
Social Function 3									-0.30***	(0.02)	-0.30***	(0.02)
Social Function 4									-0.63***	(0.03)	-0.63***	(0.03)
Social Function 5									-0.81***	(0.08)	-0.81***	(0.05)
Pain 2									-0.02	(0.01)	-0.02	(0.01)
Pain 3									-0.08***	(0.02)	-0.08***	(0.02)
Pain 4									-0.19***	(0.02)	-0.19***	(0.02)
Pain 5									-0.32***	(0.05)	-0.31***	(0.04)
Mental Health 2									-0.13***	(0.01)	-0.13***	(0.01)
Mental Health 3									-0.40***	(0.02)	-0.40***	(0.02)
Mental Health 4									-0.94***	(0.03)	-0.94***	(0.02)
Mental Health 5									-1.82***	(0.09)	-1.82***	(0.05)
Vitality 2									-0.04	(0.03)	-0.04	(0.03)
Vitality 3									-0.20***	(0.03)	-0.20***	(0.03)
Vitality 4									-0.43***	(0.03)	-0.43***	(0.03)
Vitality 5									-0.67***	(0.04)	-0.66***	(0.04)
**Model statistics**												
Cragg-Donald			192.1				153.8				192.2	
Anderson			382.2				299.0				382.4	
Endogeneity test			5.8				5.5				4.9	
BIC	236,338		236,538		234,751		234,933		230,943		231,104	
Observations	85,433		85,433		85,433		85,433		85,433		85,433	
**CIV in €**	80,522		28,130		58,083		20,266		79,037		27,869	
Physical Function									3,729		1,337	
Role Function									3,024		1,060	
Social Function									16,264		5,749	
Pain									6,316		2,218	
Mental Health									36,361		12,819	
Vitality									13,343		4,685	

Source: Own calculations based on SOEP Waves 2002-2018. *Note:* * $p < 0.10$, ** $p < 0.05$, *** $p < 0.01$. BIC Bayesian information criteria. Baseline specification without imputation of SF-6D utilities. Sum score with range from 0 to 1.

A3 Details on Instrumental Variable Creation and Regression

Lacking information on financial-worsening events, as used by Huang et al. (2018), we explored alternative instruments which have previously been applied with SOEP data: These related to future income shocks (Bayer & Juessen, 2015), or industry wage structure (Luechinger, 2009; Pischke, 2011). As our base model already included lagged income, we followed Luechinger (2009), using predicted labour income based on industry/occupation as instrument. The model outlined in equation 7 was run separately for East and West Germany to account for the persisting income and labour market differences. Deviating from Luechinger (2009), who predicted labour earnings for around 5,000 industry occupation cells, we followed Pischke (2011) and collapsed the number of industry branches and occupation groups to 33 and 22, forming a total of 726 theoretically possible industry-occupation cells. We follow the definition by Pischke (2011) as opposed to Luechinger (2009) due to concerns related to the sample-size within these industry-occupation cells being very low when following the more granular breakdown into 5,000 groups. In practice, however, we only observe 456 actually populated cells as not all occupations can be found in all industries. Each cell has on average 231 individual-year observations. For 50% of industry-occupation groups the number of individual-year observations within each cell is ≤ 40, constituting however only a total of $\approx 2,200$ individual-year observations. The full list of industries and occupations included and the mean and variance of reported incomes over the period of 2002-2018 are depicted in Appendix Figures A3.1 and A3.2.

Figure A3.1: Average Monthly Net Labour Income by Industry (2002-2018)

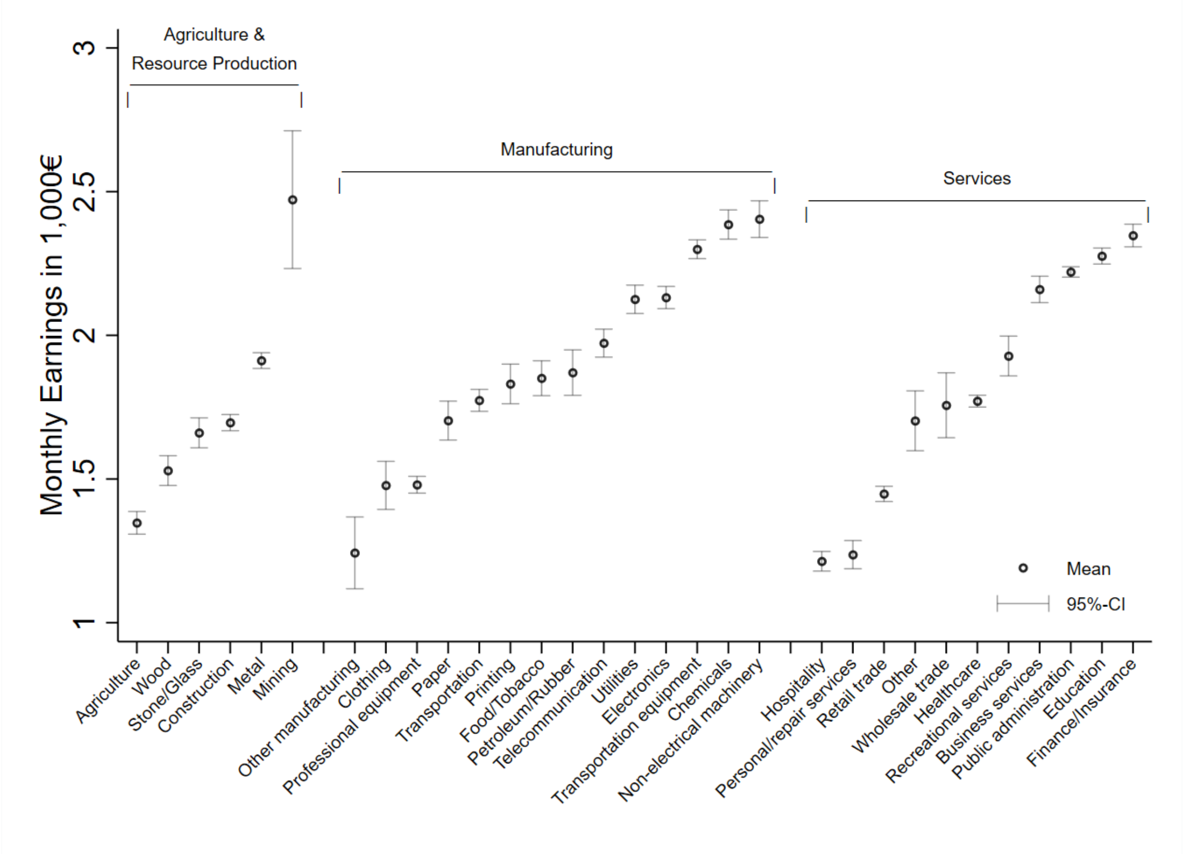

Source: Own calculations based on SOEP Waves 2002-2018. *Note:* Depicted incomes are based on hourly earnings and calculated for full time employment (40 hours per week).

Figure A3.2: Average Monthly Net Labour Income by Occupation (2002-2018)

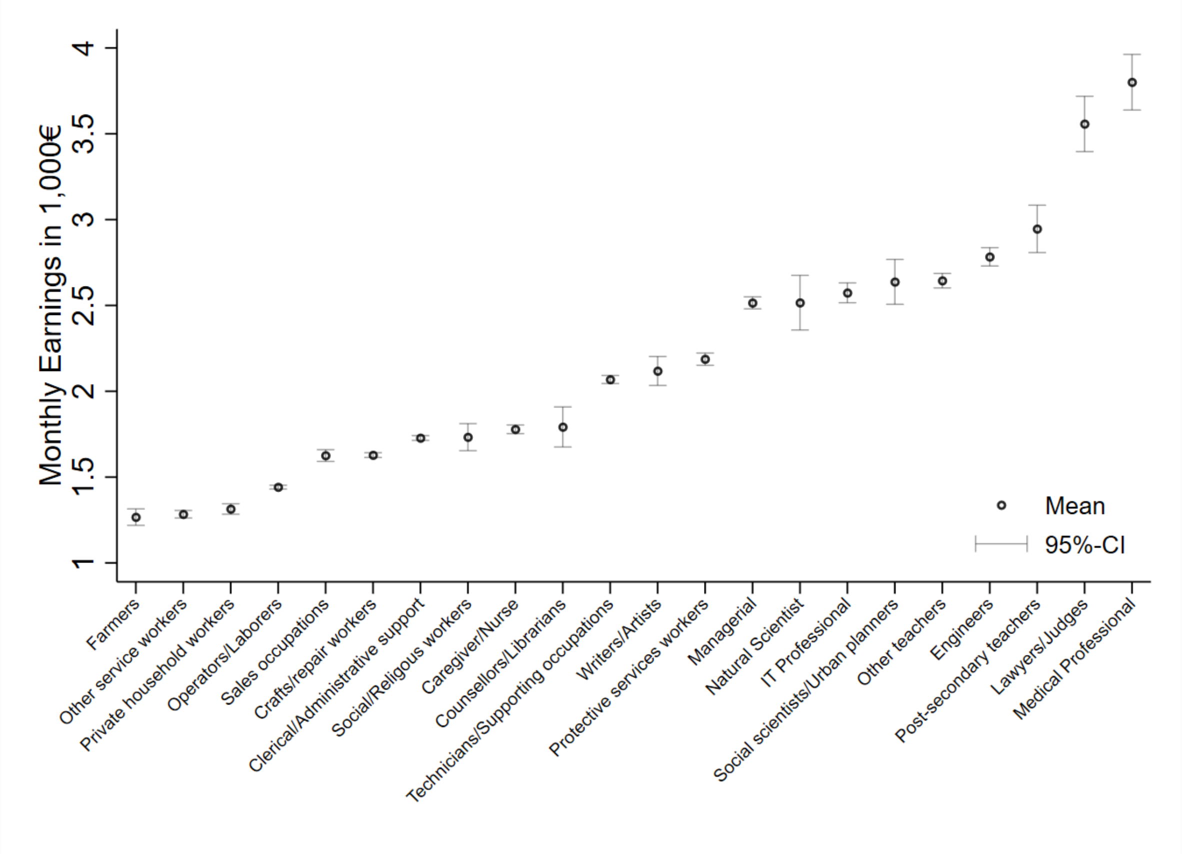

Source: Own calculations based on SOEP Waves 2002-2018. *Note:* Depicted incomes are based on hourly earnings and calculated for full time employment (40 hours per week).

Figure A3.3: Impact of industry on labour income

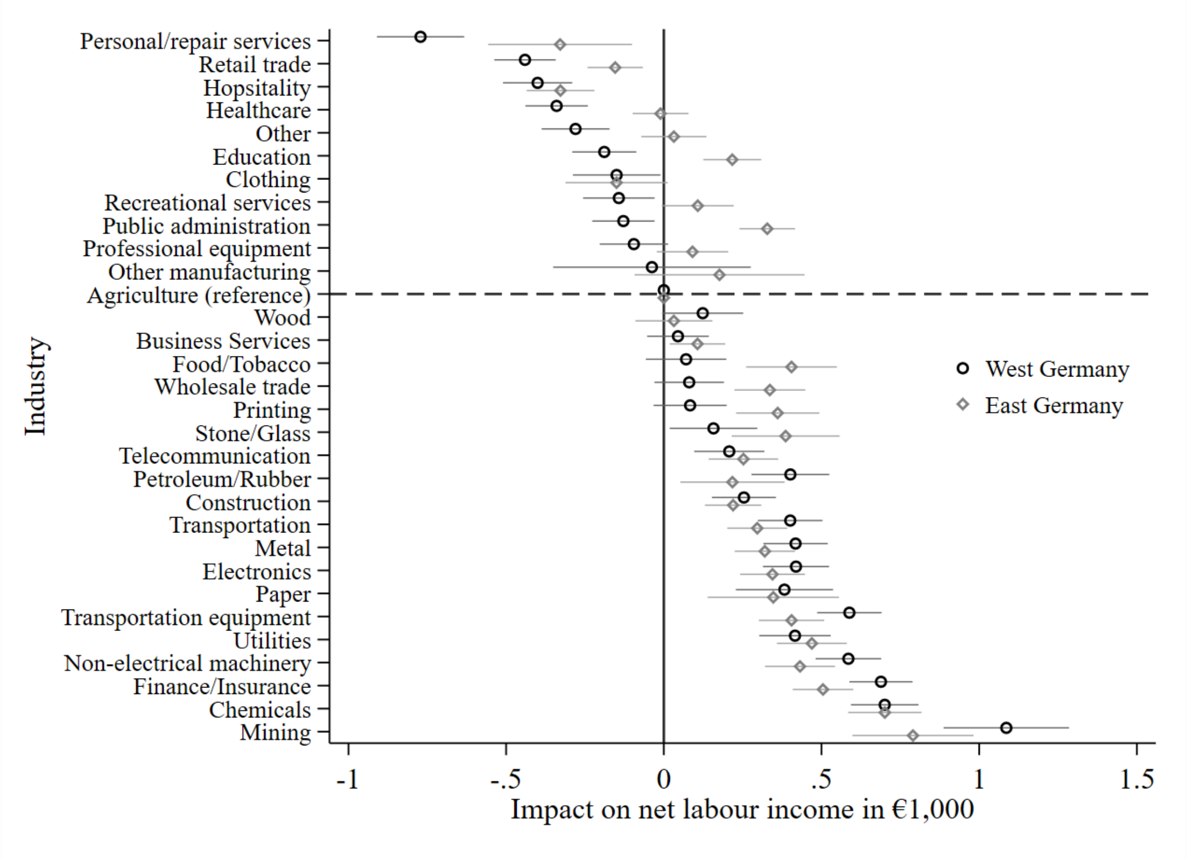

Source: Own calculations based on SOEP Waves 2002-2018. *Note:* Occupation, year and state coefficients omitted from output.

The number of observations per industry varied from 105 (Other manufacturing) to 17,230 (healthcare), with a mean of 4,040 (median 2,409). The corresponding range per occupations was 196 (Counsellors/Librarians) to 22,345 (Clerical/Administrative support) with a mean of 5,692 (median 2,642). Figures A3.3 and A3.4 report industry and occupation coefficients from the same regressions. The figures are separated to ease within industry/occupation comparisons. The range of coefficients indicate that occupation is the more important predictor of labour income. The differences in occupation coefficients between West and East Germany likely stem from the generally lower income level in the East.

Figure A3.4: Impact of occupation on labour income

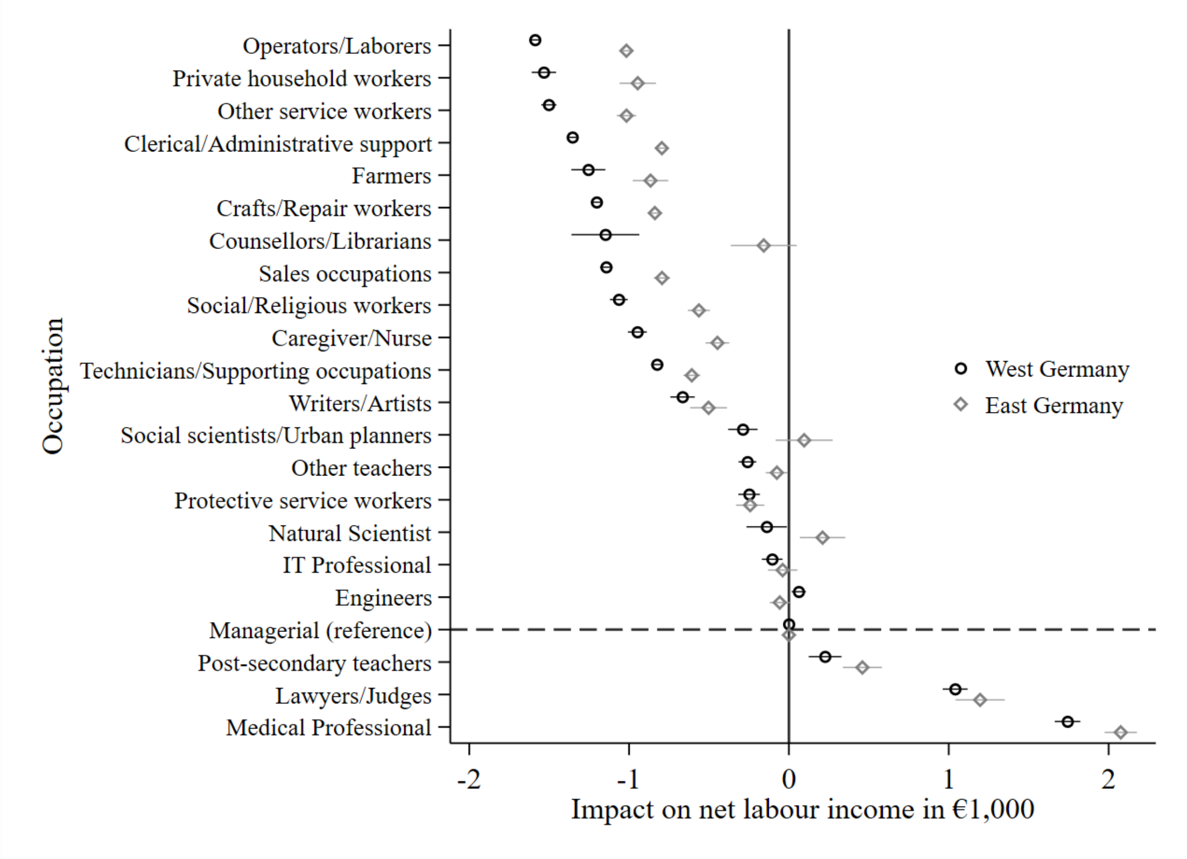

Source: Own calculations based on SOEP Waves 2002-2018. *Note:* Industry, year and state coefficients omitted from output.

Figure A3.5: QQ-plot of observed and predicted labour income

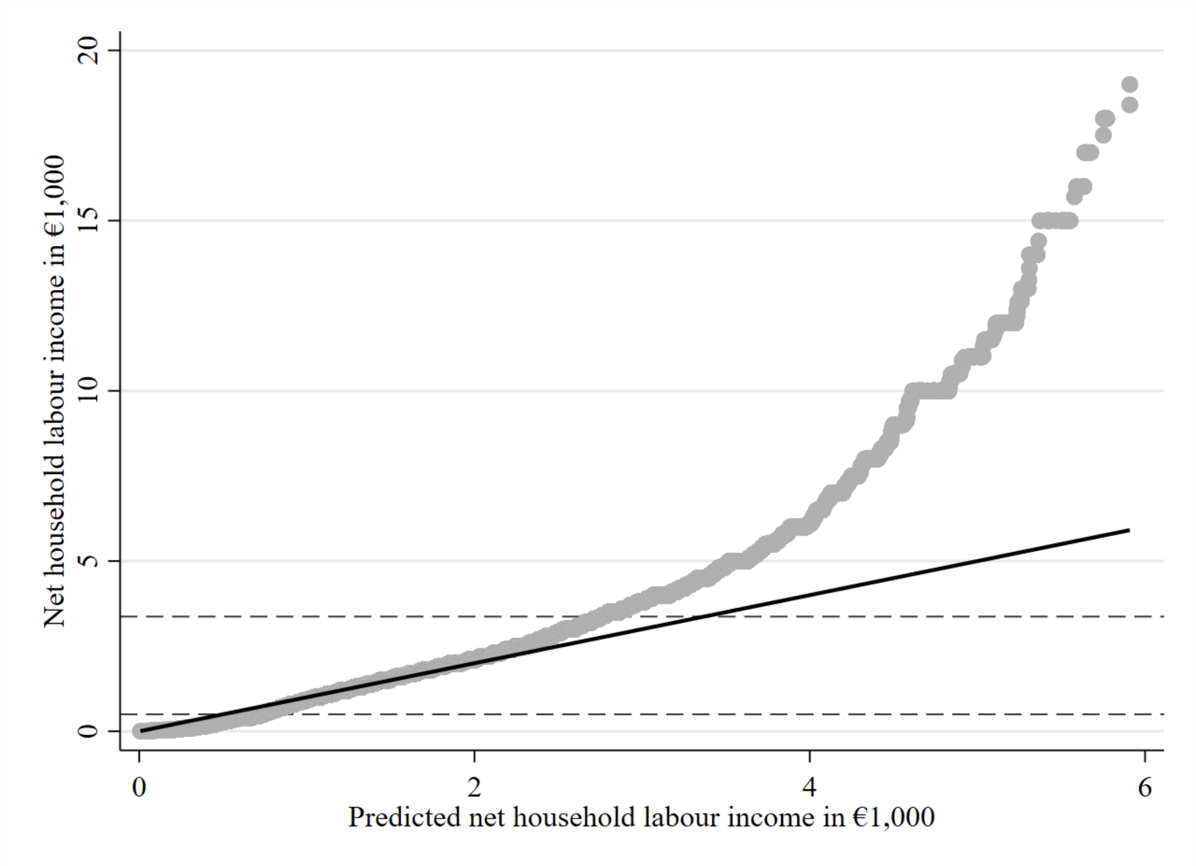

Source: Own calculations based on SOEP Waves 2002-2018. *Note:* Black line represents linear prediction with grey dots representing observed values. Dashed lines span a corridor containing 80% of the observed labour incomes. Monthly Labour incomes trimmed at €20,000.

Our income instrument is based on predicting net household labour income based on the industry and occupation individuals are employed in. The quantile-quantile plot presented in Figure A3.5 shows that the accuracy of the prediction is very high for the largest part of the labour income distribution. However, the prediction error is increasing for very high incomes, which occur less frequently.

Table A3.1: First stage results of IV regression

	Endogenous regressors			
	Income		Lagged income	
Predicted household labour income	0.250***	(0.004)	-0.006	(0.004)
Predicted household labour income ($t-1$)	0.041***	(0.004)	0.292***	(0.004)
SF-6D utility	0.089***	(0.027)	0.039	(0.028)
SF-6D utility ($t-1$)	0.034	(0.027)	0.092***	(0.028)
Disability	0.001	(0.008)	-0.010	(0.009)
Age	0.091***	(0.007)	0.076***	(0.008)
Age squared	-0.000***	(0.000)	-0.000***	(0.000)
(de facto) Married	0.075***	(0.008)	0.078***	(0.008)
Primary education	0.351***	(0.038)	0.280***	(0.040)
Tertiary education	0.028	(0.024)	0.023	(0.025)
Leisure time	0.008***	(0.002)	0.002	(0.002)
Leisure time squared	-0.001***	(0.000)	-0.000*	(0.000)
Unemployed	0.048***	(0.010)	0.032***	(0.010)
Work hours	0.004***	(0.000)	0.001***	(0.000)
Tenure	0.001	(0.000)	0.003***	(0.000)
Observations	186,902		186,902	
F-test of excluded instruments	2,837.9		3,641.2	

Source: Own calculations based on SOEP Waves 2002-2018. *Note*: * $p < 0.10$, ** $p < 0.05$, *** $p < 0.01$. Year and state dummies excluded from output.

The first stage results of the baseline IV regression shows the strong correlation between the endogenous regressors and our constructed income instruments (predicted labour income and its lag).

Figure A3.6: Impact of industry on life satisfaction

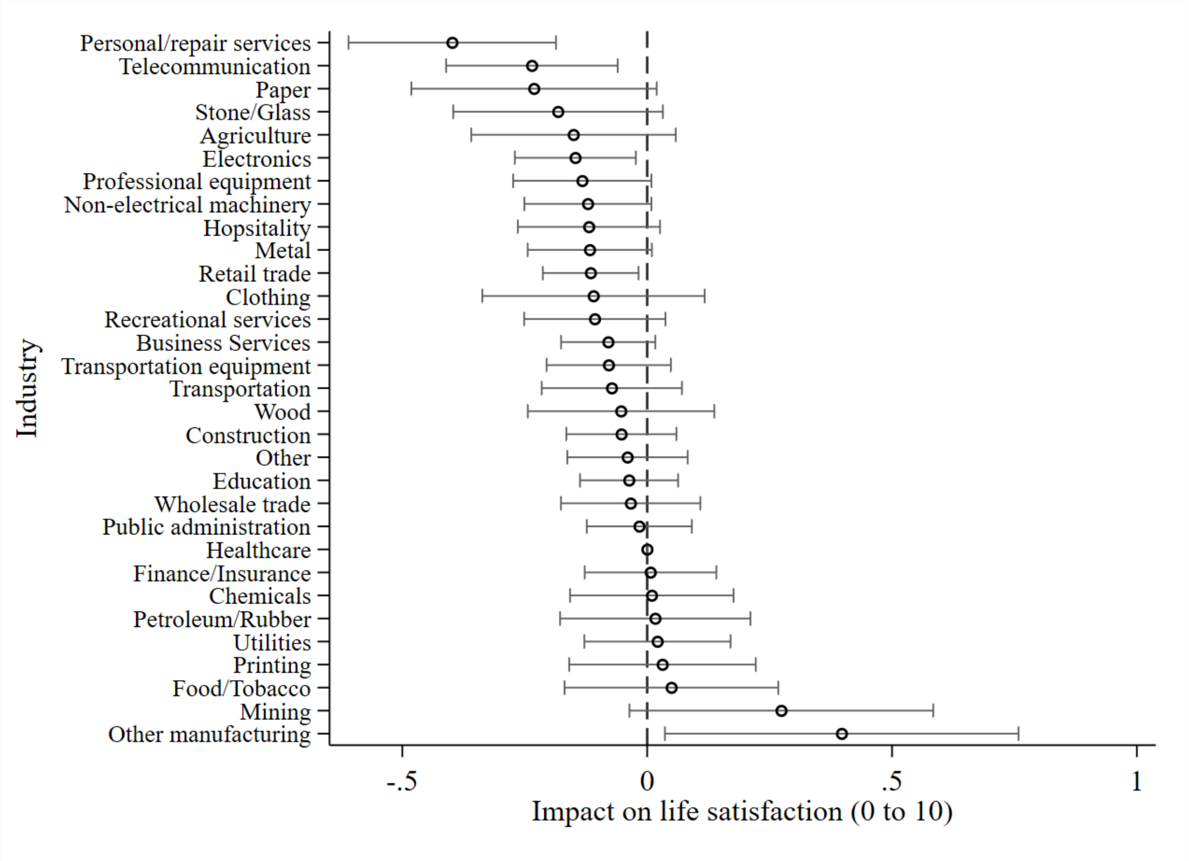

Source: Own calculations based on SOEP Waves 2002-2018. *Note:* Industry coefficients from regression of on life satisfaction controlling for income and the covariates used in the main analysis. Industry with most observation as reference category (Healthcare).

A further threat to the validity of our instrument is that industries and occupations, on which our instruments are based on, should ideally not be correlated with our dependent variable, life satisfaction. While we cannot entirely refute this, Figures A3.6 and A3.7 show that the impact of industry and occupation is modest and mostly insignificant.

Figure A3.7: Impact of occupation on life satisfaction

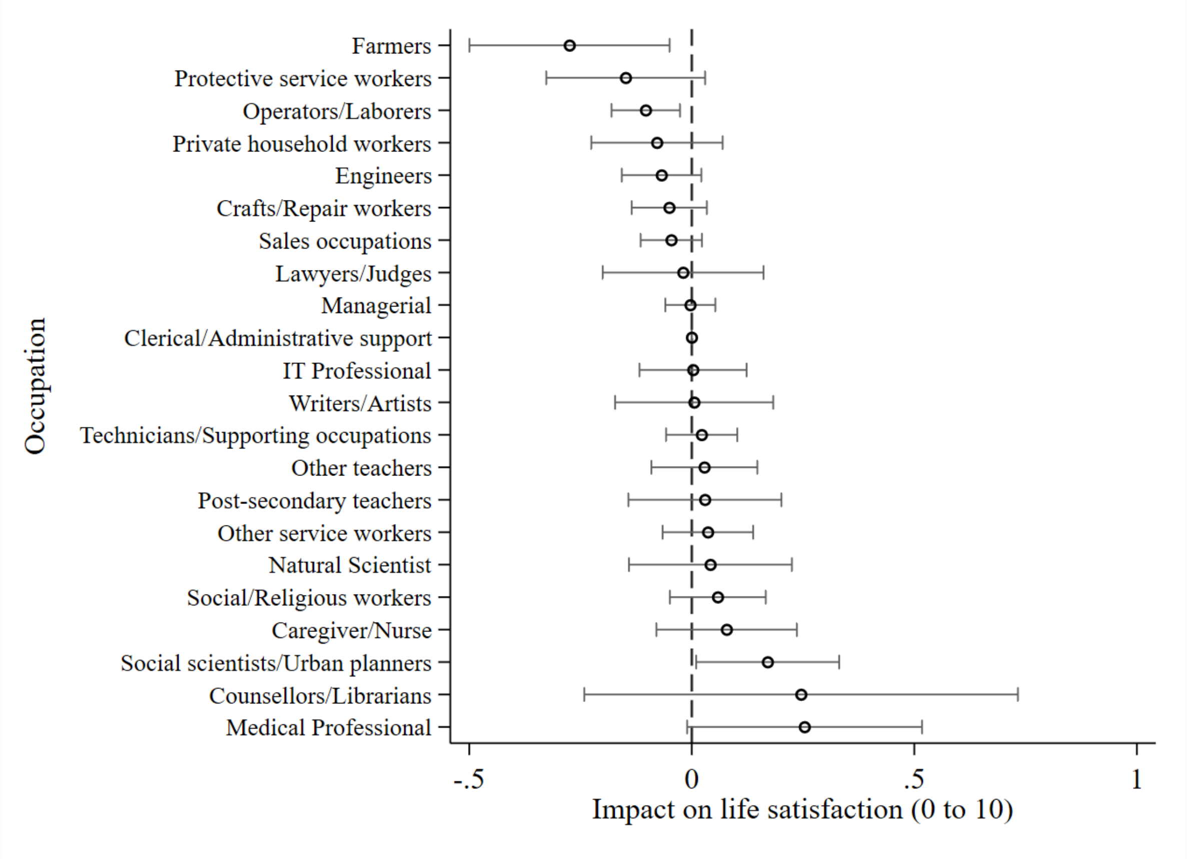

Source: Own calculations based on SOEP Waves 2002-2018. *Note:* Occupation coefficients from regression of on life satisfaction controlling for income and the covariates used in the main analysis. Occupation with most observations as reference category (Clearical/Administrative support).

A4 Health State Dependence

We explored the potential relevance of the health state dependence of consumption utility on the estimation of CIV_{QALY} values by constructing a sub-sample of individuals transitioning between good and bad health "health change sampl"). To do so, we used the mental and physical SF-12 component scores as a universal health-state measure. The mental (MCS) and physical (PCS) component scores range from 0 (worst) to 100 (best) and are normalized to have a mean of 50 and a standard deviation of 10 (Ware et al., 1995). In a first step, we calculated for each individual i their respective maximum and minimum reported mental ($MCS_i^{min/max}$) and physical ($PCS_i^{min/max}$) component score across periods. Individuals were included in the sample if they experienced an overall score difference of at least 10 ($MCS_i^{max} - MCS_i^{min} \geq 10$ and/or $PCS_i^{max} - PCS_i^{min} \geq 10$). Subsequently, we calculated the mean score ($\overline{MCS_i}$ and $\overline{PCS_i}$) for each individual. A health state for a given individual in period t was considered good if both mental and physical scores were greater or equal to the mean ($MCS_{it} \geq \overline{MCS}_i$ and $PCS_{it} \geq \overline{PCS}_i$), and bad if both were below ($MCS_{it} < \overline{MCS}_{it}$ and $PCS_i < \overline{PCS}_i$). Lastly we conditioned on the consecutive observation of health states with at least two periods spend in each to allow for a fixed effects estimation for good and bad health states separately. This reduced our sample considerably to 5,112 individuals. Table A4.1 compares the summary statistics for the main analysis sample and the health change sample.

Table A4.1: Descriptive statistics - health state dependence sample

	All Individuals		Health Change Sample	
**Variable**	**Mean**	**Std. Dev.**	**Mean**	**Std. Dev.**
Life satisfaction	7.09	1.71	6.87	1.75
Income in 1000's	2.03	1.29	1.97	1.12
SF-6D utility	0.73	0.13	0.70	0.13
Disability	0.14	0.35	0.17	0.37
Age in years	53.67	15.78	56.33	15.51
(de facto) Married	0.67	0.47	0.68	0.47
Education: Primary	0.12	0.32	0.12	0.32
Education: Tertiary	0.63	0.48	0.65	0.48
Education: Secondary	0.25	0.43	0.23	0.42
Leisure time	2.18	2.03	2.35	2.10
Employed	0.56	0.50	0.50	0.50
Unemployed	0.04	0.21	0.04	0.21
Work hours	21.22	20.99	18.91	20.83
Tenure	7.03	9.96	6.59	9.95
Observations		186,902		48,861
Individuals		29,735		5,112

Overall there was a good overlap between the sample characteristics of the full analysis sample and the health change sample. The notable exceptions were slightly lower levels of life-satisfaction, income and SF-6D utility values and a slightly higher average age and disability-rate within the health change sample. These slight differences in age- and health-related variables were not surprising as we conditioned on individuals experiencing a substantial health change.

Figure A4.1 provides an overiew on the two most important variables for the CIV_{QALY} estimation; health and income. Panel (a) depicts the distribution of SF-6D utilities within the health change sample (black) and the rest of the sample (grey). Panel (b) depicts health utilities across health states within the health change sample with good health in grey and bad health in black. It becomes clear that our definition of bad health (based on MCS and PCS) coincides with substantially lower health utilities with the mean falling from 0.74 in good health to 0.61 in bad health.

Figure A4.1: Health change sample overview

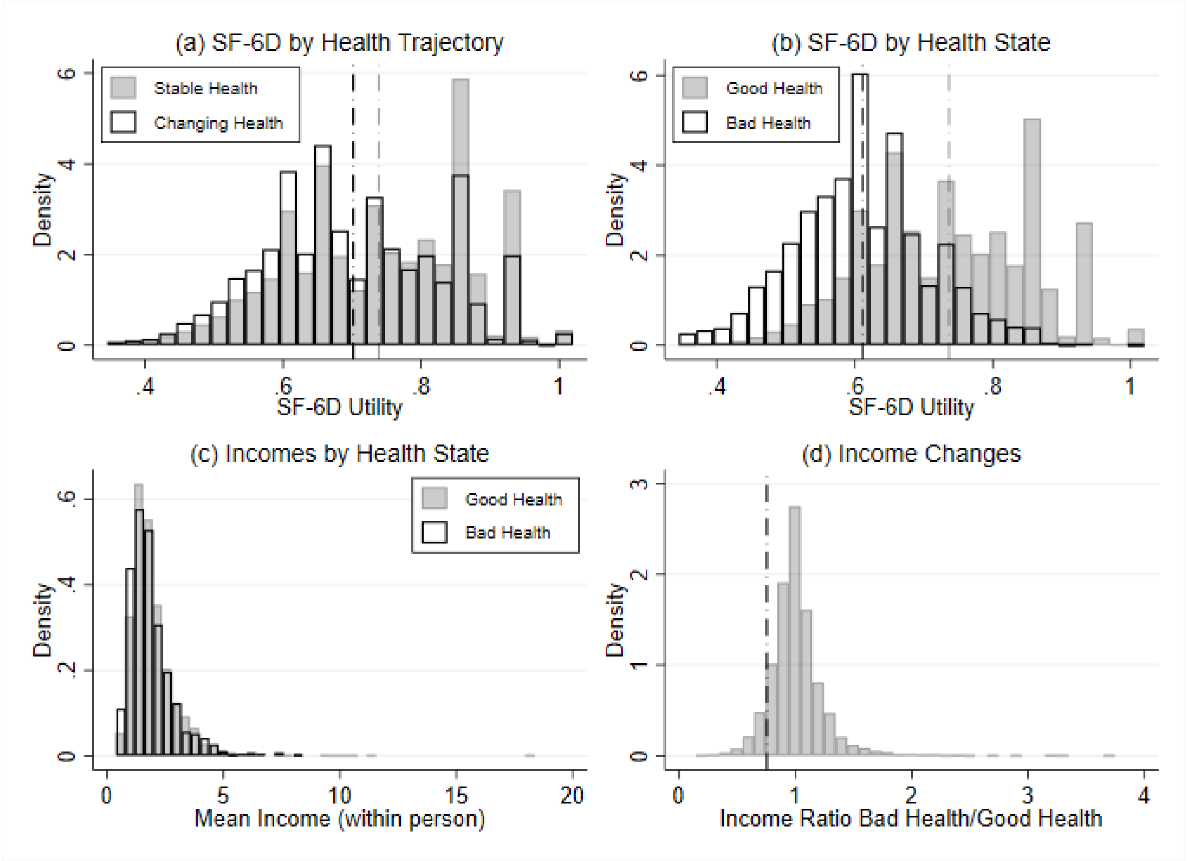

Source: Own calculations based on SOEP Waves 2002-2018. *Note:* Panel (a) depicts distribution of SF-6D utility values and their means (dash-dotted lines) for individuals without a 10-point component score change over the observation period and individuals in the health-state dependence sample. Panel (b) depicts the distribution of SF-6D utilities of people in the health state dependence sample in their respective good and bad health states. Panel (c) plots the distribution of within-person mean equivalized household incomes in good and bad health states. Panel (d) depicts the ratio of equivalized housholed income in bad and good health states with the dash-dotted line marking the lower one standard-deviation.

As mentioned in the main body of the text, one concern when studying health state dependence of consumption utility is that there is the possibility that lower health is associated with a decrease in income leading to larger income coefficients. Figure A4.1 Panel (c) illustrates that this concern is warranted by plotting the within-person mean incomes across health states for the working population showing a shift of the distribution towards lower incomes.

Finkelstein et al. (2013) and Kools and Knoef (2019) addressed this directly using a two-step procedure to obtain income coefficients for individuals with stable incomes across health states. Our fixed-effects based approach does not allow for this. Instead we calculated the ratio between within-person income means in good and bad health states and excluded those individuals whose income-ratio was less than one standard deviation below the mean income-ratio, corresponding

to an income drop of 25% and more. Panel (d) plots these ratios and the corresponding cut-off point. This removes approximately 10% of working individuals. Table A4.2 presents the estimation results when excluding individuals with high income differences, which left a total of 4,656 observations.

Table A4.2: Health state dependence - excluding high income losses

	**Baseline**		**Good Health**		**Bad Health**	
	OLS	IV	OLS	IV	OLS	IV
Income in 1000's	0.07***	0.16**	0.06***	0.12	0.06	0.12
	(0.02)	(0.08)	(0.02)	(0.09)	(0.04)	(0.25)
Income in 1000's ($t-1$)	0.01	-0.01	0.02	0.00	0.03	0.06
	(0.01)	(0.07)	(0.02)	(0.07)	(0.03)	(0.21)
SF-6D utility	3.54***	3.54***	2.44***	2.43***	4.11***	4.09***
	(0.11)	(0.10)	(0.14)	(0.13)	(0.39)	(0.39)
SF-6D utility ($t-1$)	0.07	0.08	0.16	0.16	0.34	0.34
	(0.11)	(0.10)	(0.13)	(0.12)	(0.27)	(0.27)
**Model statistics**						
Cragg-Donald		575.5		415.9		81.6
Anderson		1,120.0		808.8		160.6
Endogeneity test		1.7		0.6		0.1
BIC	137,041	137,087	93,643	93,662	34,344	34,349
Observations	44,667	44,667	32,389	32,389	12,278	12,278
**CIV in €**	50,571	25,346	32,347	20,626	51,034	24,462

Source: Own calculations based on SOEP Waves 2002-2018. *Note:* * $p < 0.10$, ** $p < 0.05$, *** $p < 0.01$. BIC Bayesian information criteria.

In a second step we further restricted the sample to only those individuals who worked during their respective participation in the panel. This was motivated by the fact that individuals might have moved into early retirement or unemployment following health changes, thereby decreasing the within-person income variance across periods. Table A4.3 depicts the estimation results after restricting the sample to the 3,032 individuals without large negative income changes and working throughout the entire observation period.

Table A4.3: Health State Dependence - excluding high income losses & unemployed/retired

	**Baseline**		**Good Health**		**Bad Health**	
	OLS	IV	OLS	IV	OLS	IV
Income in 1000's	0.07***	0.11	0.06***	0.04	0.08*	0.21
	(0.02)	(0.08)	(0.02)	(0.09)	(0.04)	(0.24)
Income in 1000's $(t-1)$	0.00	0.02	0.02	0.04	0.02	0.04
	(0.02)	(0.06)	(0.02)	(0.07)	(0.03)	(0.21)
SF-6D utility	3.45***	3.45***	2.51***	2.51***	3.52***	3.48***
	(0.13)	(0.12)	(0.17)	(0.15)	(0.46)	(0.46)
SF-6D utility $(t-1)$	0.08	0.09	0.23	0.23	0.41	0.42
	(0.13)	(0.12)	(0.15)	(0.14)	(0.32)	(0.33)
**Model statistics**						
Cragg-Donald		454.9		331.8		65.4
Anderson		880.9		642.1		128.4
Endogeneity test		0.5		0.1		0.3
BIC	89,392	89,401	61,756	61,758	21,876	21,892
Observations	29,508	29,508	21,530	21,530	7,978	7,978
**CIV in €**	46,735	28,776	37,299	36,176	41,513	15,599

Source: Own calculations based on SOEP Waves 2002-2018. *Note:* * $p < 0.10$, ** $p < 0.05$, *** $p < 0.01$. BIC Bayesian information criteria.

In a last step we restrict our sample to individuals experiencing more severe and sudden health changes. Given our selection on a 10-point decrease in mental or physical component scores the sample might include individuals who experienced a gradual decrease in their health, which is substantial in absolute terms, but occurred over a long time-period. In such cases, the definition of health states based on the overall within-person mean wrongfully identifies individuals as switching between different health states despite the actual differences between both being rather small. Figure A4.2 plots the differences in mean mental $(\overline{MCS}_i^{good} - \overline{MCS}_i^{bad})$ and physical $(\overline{PCS}_i^{good} - \overline{PCS}_i^{bad})$ component scores and the raw distribution of differences for each health dimension for all 5,112 individuals. By construction higher score changes indicate worse health changes. The mean difference in mental health scores was 7.90 and for physical health 6.46, however there was a substantial number of individuals who experienced small health differences between both states.

Figure A4.2: Health change sample - mental/phsyical component score differences

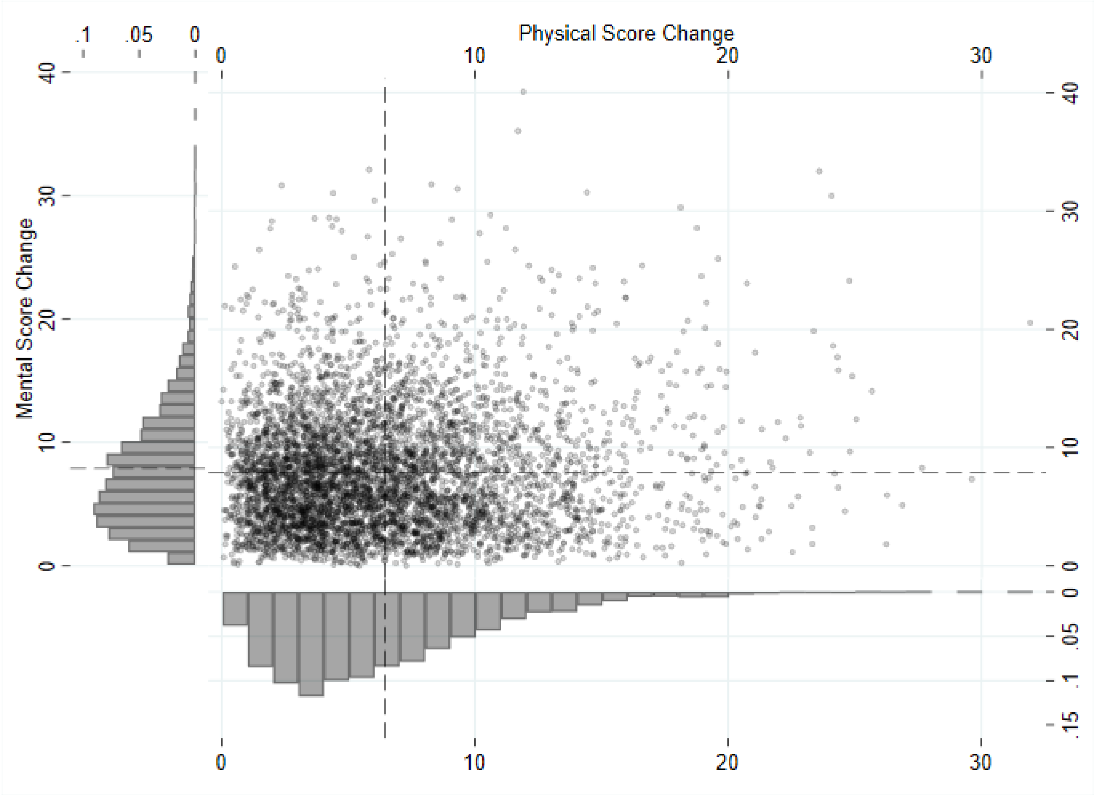

Source: Own calculations based on SOEP Waves 2002-2018
Note: The main panel plots the individual-level differences between mean mental (vertical axis) and physical (horizontal axis) component score changes between good and bad health states. The raw distributions are plotted in the vertical and horizontal side panels with black dashed lines indicating mean score changes.

We explored to what extend the fact that some individuals in the health change sample only experienced a small or gradual health change across defined health states by excluding individuals for which the differences in mean component scores in both dimension was below 5 or one half standard deviation. Table A4.4 depicts the results when also excluding these individuals next to those with high income losses as well as consistently out of work, leaving only 2,567 individuals in the analysis sample. As expected the point estimates for the IV specification become very imprecise. Nonetheless, the general pattern indicating higher income coefficients in bad health states remains across specifications, suggesting the presence of positive health state dependency.

Table A4.4: Health State Dependence - Excluding high income losses, unemployed/retired & only severe health changes

	**Baseline**		**Good Health**		**Bad Health**	
	OLS	IV	OLS	IV	OLS	IV
Income in 1000's	0.07***	0.13	0.05**	0.06	0.08*	0.24
	(0.02)	(0.09)	(0.02)	(0.10)	(0.05)	(0.28)
Income in 1000's $(t-1)$	-0.01	0.01	0.00	0.02	-0.00	0.11
	(0.02)	(0.07)	(0.02)	(0.08)	(0.03)	(0.21)
SF-6D utility	3.64***	3.64***	2.64***	2.64***	3.65***	3.62***
	(0.14)	(0.13)	(0.19)	(0.17)	(0.49)	(0.50)
SF-6D utility $(t-1)$	0.05	0.06	0.22	0.22	0.50	0.50
	(0.14)	(0.13)	(0.17)	(0.16)	(0.34)	(0.35)
**Model statistics**						
Cragg-Donald		346.5		243.6		55.3
Anderson		672.9		473.2		108.9
Endogeneity test		1.1		0.1		0.5
BIC	74,566	74,586	50,415	50,416	19,184	19,225
Observations	24,272	24,272	17,414	17,414	6,858	6,858
**CIV in €**	68,898	27,828	54,494	35,519	54,631	11,890

Source: Own calculations based on SOEP Waves 2002-2018. *Note:* * $p < 0.10$, ** $p < 0.05$, *** $p < 0.01$. BIC Bayesian information criteria.
